# The Nanotheranostic Researcher’s Guide for Use of Animal Models of Traumatic Brain Injury

**DOI:** 10.3390/jnt2040014

**Published:** 2021-12-06

**Authors:** Brandon Z. McDonald, Connor C. Gee, Forrest M. Kievit

**Affiliations:** Department of Biological Systems Engineering, University of Nebraska-Lincoln, Lincoln, NE 68583-0726, USA

**Keywords:** neurotrauma, nanoparticle, neurobehavior

## Abstract

Traumatic brain injury (TBI) is currently the leading cause of injury-related morbidity and mortality worldwide, with an estimated global cost of USD 400 billion annually. Both clinical and preclinical behavioral outcomes associated with TBI are heterogeneous in nature and influenced by the mechanism and frequency of injury. Previous literature has investigated this relationship through the development of animal models and behavioral tasks. However, recent advancements in these methods may provide insight into the translation of therapeutics into a clinical setting. In this review, we characterize various animal models and behavioral tasks to provide guidelines for evaluating the therapeutic efficacy of treatment options in TBI. We provide a brief review into the systems utilized in TBI classification and provide comparisons to the animal models that have been developed. In addition, we discuss the role of behavioral tasks in evaluating outcomes associated with TBI. Our goal is to provide those in the nanotheranostic field a guide for selecting an adequate TBI animal model and behavioral task for assessment of outcomes to increase research in this field.

## 1. Introduction

Traumatic brain injury (TBI) is currently the leading cause of injury-related morbidity and mortality worldwide, with an estimated global cost of USD 400 billion annually [1]. Behavioral outcomes associated with TBI begin with primary injury to the brain resulting from an externally applied force [2]. These external forces can originate from direct contact between the brain and an object or through non-impact situations including rotational acceleration and the energy waves produced from blasts [3,4]. This can result from falls, motor vehicle accidents, assault, domestic violence, military warfare, and even recreational sports including football, hockey, and boxing [2]. These multiple mechanisms of impact generate a broad spectrum of injury severities and behavioral outcomes, leading to difficulties in developing diagnostic and prognostic protocols, let alone effective treatments. Thus, there is still no approved therapy that has shown efficacy in reducing the long-term secondary effects following TBI.

TBI patients have a 2–4-fold increase in the risk of developing dementia later in life due to even a single instance of TBI followed by a loss of consciousness (LOC) [5]. In conjunction with aging, individuals who have experienced mild TBI are at increased risk for developing Alzheimer’s disease, at 2.3 and 4.5 times more likely for moderate and severe TBI, respectively [6]. Even repeated mild injuries, such as those among retired professional American football players, have been correlated to long-term cognitive deficits. Retired players who had suffered three or more concussions in their careers had a 5-fold increase in mild cognitive impairments compared to their counterparts with no history of concussions [5]. Additionally, Parkinson’s disease, amyotrophic lateral sclerosis (ALS), Creutzfeldt–Jakob disease, and chronic traumatic encephalopathy (CTE) were also all found to be associated with the progression of chronic TBI [5]. Due to the association of TBI with these progressive neurodegenerative diseases, viable treatment options must be developed with an in-depth knowledge of the injury’s pathophysiology, lest the current therapeutic stalemate continue.

Several safety precautions have been implemented to prevent head trauma, including the provision and advancement of helmets, seatbelts, and airbags. However, the major problem facing TBI patients is the spread of secondary corrosive damage to the surrounding brain tissue following this initial impact. This lethal progression of secondary damage is caused by a disruption in the oxidant/antioxidant equilibrium of the brain, which forces a biochemical imbalance, leading to chronic oxidative stress [7]. Oxidative stress leads to the damage of lipids, proteins, and DNA in the brain and creates deterioration similar to the development of some neurodegenerative diseases [7]. Oxidative stress progresses alongside a variety of other biochemical malfunctions, including glutamate toxicity in neurons, mitochondrial dysfunction, and blood–brain barrier (BBB) disruption [8]. Due to this secondary damage, TBI presents with a multitude of physical, cognitive, and behavioral deficits. However, the evolution of these deficits is highly variable and can range from minor concussive symptoms to severe TBI, leading to probable death.

Unfortunately, differences among patients and their injuries provide a variety of complications for medical personnel in determining efficient diagnoses and effective treatments. From 1993 to 2016, there were 30 failed clinical trials involving various forms of treatment [9]. These treatment options included temperature control, hypertonic saline, progesterone, prostacyclin, surgical intervention, intracranial pressure monitoring, and various pharmacological therapeutics [9]. Although there has been success in Phase II trials, all these treatments have failed during larger, multi-center Phase III trials. These failures have resulted due to a variety of problems during testing for the efficacy of treatments. Progesterone for the Treatment of Traumatic Brain Injury (ProTECT) and Study of Neuroprotective Agent, Progesterone, in Severe Traumatic Brain Injury (SyNAPse) both resulted in negative outcomes during Phase III trials [10]. Researchers postulate that these failures were the result of suboptimal dosing during Phase II trials, suggesting inadequate delivery into the brain and poor target engagement, in addition to heterogeneity between injuries [10]. Other clinical trials have had similar issues, including problems with clinical trial design, lack of accurate injury phenotyping, and inadequate outcome assessment tools [11]. Injury heterogeneity and inadequate outcome assessment tools are capable of being mitigated with effective classification systems. Classification systems have been previously constructed for categorizing the injury severity of TBI in humans immediately following diagnostic exams from medical professionals. Initial methods for classifying TBI in a clinical setting are efficient, but simplistic in approach, leaving room for error between different degrees of human injury. However, recent literature has investigated the most important variables for assessing TBI in the hopes of improving upon the original designs to create a more effective classification system [12,13].

While methods for classifying degrees of injury in humans have advanced, efforts have also been directed towards developing animal models for TBI to provide an effective comparison to human injuries [14,15]. These models have been used to understand the pathophysiological mechanism for the progression of different degrees of TBI. Additionally, animal models have aided in the development of potential treatments for the reduction of oxidative stress, BBB dysfunction, and various other biochemical impairments [8,14]. Recently, Operation Brain Trauma Therapy (OBTT) was developed as a multi-center, preclinical consortium to identify therapies that are beneficial in alleviating damage from head trauma in animal models [11]. The OBTT makes use of several animal models in three distinct injury categories, focal, diffuse, and non-impact injury, creating a broad spectrum of potential pathophysiological outcomes [2,14]. Each model has unique procedures and outcomes in the hopes of providing a sufficient translation to the variety of head traumas that occur in humans. Through these models, comparisons can be derived between the various degrees of human injury severity, which will ultimately lead to improvements in diagnostics and treatment protocols.

Additionally, these animal models can be used in conjunction with behavioral assessments to identify the cognitive outcomes associated with different mechanisms of injury. These behavioral tasks have been established to address a variety of neurological changes associated with TBI, including deficits in spatial and non-spatial memory. Additionally, impact to specific regions of the brain or spread of secondary injury could result in emotional impairment and deficits in motor coordination, both present in clinical presentations of TBI. In general, we see most of these deficits across all models; however, behavioral outcomes are highly correlated with levels of injury severity, and repeated injuries result in variable changes in behavior [16]. While not being covered in this review, sex also has a profound effect on TBI behavioral outcomes and may play a role in the pathophysiology of TBI [17]. Choosing the best behavioral paradigms to study preclinical models is an important task; thus, we provide information on a variety of tasks in different categories to best assess novel nanotheranostics to try and accelerate clinical success.

Animal models and behavioral assessments provide varying strengths and weakness depending on the mechanism of injury and associated cognitive deficits in both acute and chronic stages of injury progression. Therefore, this review aims to provide guidelines for assessing therapeutics by investigating the role of animal models and behavioral tasks for evaluating TBI. Primary objectives for this review include: (1) evaluating different classification methods used for categorizing levels of TBI injury severity in a clinical setting; (2) characterizing TBI animal models based on their strengths, weaknesses, and previously completed experiments; (3) characterizing behavioral tasks based on their association with neurological deficits; and (4) providing an effective comparison between clinical presentations of TBI and animal models based on mechanism of injury and pathophysiological consequences. It is hoped that this review will ease the transition of nanotheranostics researchers into the neurotrauma field, where novel treatment and diagnostic strategies are urgently needed. For those looking for the state of nanotheranostics in the TBI field, we recommend these recent reviews: [18–20].

## 2. Classification of TBI Injury Severity in Humans

The severity of a patient’s TBI is primarily affiliated with the mechanism of injury in which the initial applied force is delivered to the head. This force will drive the secondary progression of damage and can provide valuable insight into the overall development of the condition. However, there are several additional variables that are required to effectively characterize a patient’s level of injury. These factors help determine the overall injury progression of the individual. While a patient’s injuries can range from mild, presenting with concussive symptoms, to severe, leading to probable death, the classification methods developed by previous literature have determined the different categories of human TBI in between these broad outcomes.

### 2.1. Glasgow Coma Scale

Initial analysis for categorizing the behavioral deficits following TBI in a clinical setting is based on the Glasgow Coma Scale (GCS), originally developed in 1974 [21,22]. Although the classification criteria for this system was developed nearly 50 years ago, the system is still regularly used by medical professionals to evaluate the degree of injury immediately following head trauma. The GCS provides a reference score calculated following an examination from a medical professional to identify the strength of a patient’s response in three main areas: eye movement, verbal response, and motor function (scale shown in Table 1) [21–23]. Each category is scored based on criteria increasing in cognitive complexity from a score of 1–6. Summing the three scores allows for a better understanding of a patient’s TBI severity and enhances the ability to explore the relationships between score and outcome on an academic level. The scoring system is categorized into three sections: mild, moderate, and severe TBI. Mild injuries receive scores ranging from 13–15 and severe injuries receive scores of 3–8. The GCS system has been used for several decades due to its effectiveness in predicting outcomes of TBI. A study taking place in 1999 showed that outcome predictions made using this model were accurate 76.3% of the time at admission, 82.5% preoperatively, 77.1% at 24 h, 63.3% at 3 days, and 69.7% at 7 days post-TBI [24]. Additionally, in 2014, GCS scores obtained following patients’ exams were shown to be positively correlated with assessments of metabolism, neuroimaging, collected biomarkers, and prediction of mortality [22]. However, the GCS method suffers from limitations when predicting severe TBI outcomes. From the 1999 study, 75.8% of the overall outcome predictions were correct; however, predictions for an outcome of severely disabled were only correct 12.2% of the time [24]. It is also important to note that successful predictions for severe TBI (71.2%) were much lower than predictions of moderate (90%) and mild (92.9%) TBI [25]. Additionally, GCS scores may be impacted by a variety of circumstances including behavioral changes from drug and alcohol intoxication, misinterpretation of patients’ responses, and even early medical intervention such as intubation which can lead to inaccurate assessment from the GCS [26]. Ultimately, GCS has continued to provide value in TBI classification due to its simplicity and overall efficiency, specifically for triage while stabilizing patients. However, this method lacks the ability for an ultimate diagnostic report due to external circumstances and poor predictability for determining differences between moderate and severe TBI based on the criteria provided in the scoring system.

### 2.2. Mayo Classification of TBI

In order to build upon the GCS method and provide a more complete classification system for the evaluation of TBI injuries, in 2007, the Mayo Clinic developed a model incorporating a variety of variables, including death, LOC, post-traumatic anterograde amnesia (PTA), and computed tomography (CT) imaging [12]. Similar to GCS, each of these variables was used to help categorize injuries into three sections ranging from symptomatic TBI (possible), mild TBI (probable), and moderate–severe TBI (definite) [12]. Mayo’s method was able to improve upon the GCS method by utilizing additional details following a patient’s exam to effectively achieve a diagnosis [12]. Comparisons were evaluated between Mayo’s classification system to GCS, PTA, and LOC classifications alone for the evaluation of 1678 patients [12]. Mayo’s model was shown to identify additional patients presenting with moderate–severe TBI that other methods classified as mild due to the lack of additional parameters. Additionally, Mayo’s classification system was able to provide a category for patients with possible TBI based on symptoms that no other model was able to establish previously. Over 50% of the patient study fell into this symptomatic TBI classification, indicating that a large percentage of head trauma may not result in pronounced cognitive deficits detected by the GCS system. Individuals experiencing symptoms of TBI from concussions and minor head trauma may still require medical care, which may have been overseen from previous classification methods. Unfortunately, Mayo’s system fails to distinguish between moderate and severe TBI, which lacks details for a wide range of treatment possibilities for the medical community.

### 2.3. Collaborative European NeuroTrauma Effectiveness for Research for TBI (CENTER-TBI)

Recently, in April of 2020, analysis conducted in the Collaborative European NeuroTrauma Effectiveness Research for TBI (CENTER-TBI) expanded upon previous models for evaluating TBI injuries in humans using a wide variety of variables and characteristics [13]. Data were collected from 4509 patients across Europe and categorized into clusters using a range of five collective “building blocks”: demographics, clinical severity, secondary insults, cause of injury, and imaging characteristics, such as CT imaging or Magnetic Resonance Imaging (MRI) [13]. Variables were evaluated to determine strength of significance, where cause of injury remained the most significant determinant for the condition’s progression, followed by the presence of major extracranial injury, GCS, and imaging characteristics. Following characterization, CENTER-TBI provided four separate categories for TBI injury in humans—mild, upper intermediate, lower intermediate, and severe—and identified the likelihood of each respective outcome using the Glasgow Outcome Scale Extended (GOSE) [13]. The additional category for dividing moderate TBI is an improvement from previous classification models, allowing for additional prognostic guidance. The study also established probabilities for expected behavioral outcomes in each of the categories. The percentage of patients remaining in their previously affiliated category after resampling was 97.4%, confirming a 95% confidence interval [13]. Following this study, researchers developed a prediction model for determining an individual’s functional outcome based on the variables described previously, along with additional vitals. Researchers applied baseline admissions characteristics from examinations and a prediction of the prognostic results for a 6-month mortality time frame was collected. This prediction model represents the potential growth in the field of TBI classification. Researchers and medical personnel would be able to determine an individual’s treatment based on a handful of characteristics capable of being tested upon entry into the hospital following their initial TBI. While initial results from GCS scores are efficient and useful for providing an assessment for the urgency in treating a patient following admission to a medical facility, developing classification methods based on additional information is necessary to determine the overall progression of TBI.

## 3. Categories of TBI

TBI can often be used to describe a broad condition with varying degrees of damage, but the causal injuries associated with TBI are categorized into three distinct forms: focal, diffuse, and non-impact. Focal injuries in a human population are created through direct impact forces acting on the skull, which causes compression of the underlying tissue. Focal injuries include skull fractures, contusions, lacerations, hemorrhages, and subdural, epidural, and intraparenchymal hematomas [27]. Contusions from focal injuries are often due to penetrating impacts or severe blunt force trauma, differing from other ailments that may be caused by diffuse injury. Contusions can occur in two different forms: coup, also known as ipsilateral, or contrecoup contusions [27]. Coup contusions occur below the impact site when the head absorbs impact, and contrecoup contusions occur opposite of the impact site. For example, impact forces applied to the frontal lobe (hitting head against wall) produce contrecoup contusions near the occipital lobe. Contusions differ from lacerations simply by the forces causing the injury, as contusions are caused by direct blunt forces while lacerations are caused by shearing forces placed upon the tissue [27]. Additionally, contusions are associated with damage to small blood vessels, while hemorrhaging is associated with bleeding in the subarachnoid or subdural space. Subarachnoid hemorrhaging may result from either focal or diffuse injuries but is more often seen in diffuse injuries [28]. Subdural hematomas are usually caused by ruptured veins due to quick acceleration and deceleration forces [28]. A concern with focal injuries is intracerebral hematomas, which can develop over 24 h following contusions, and, specifically, the subset of intracerebral hematomas that develop with a delayed onset 1 to 3 days after TBI. Delayed intracerebral hematomas are incredibly dangerous, with a mortality rate between 50% and 75% [27].

**Figure 1.**
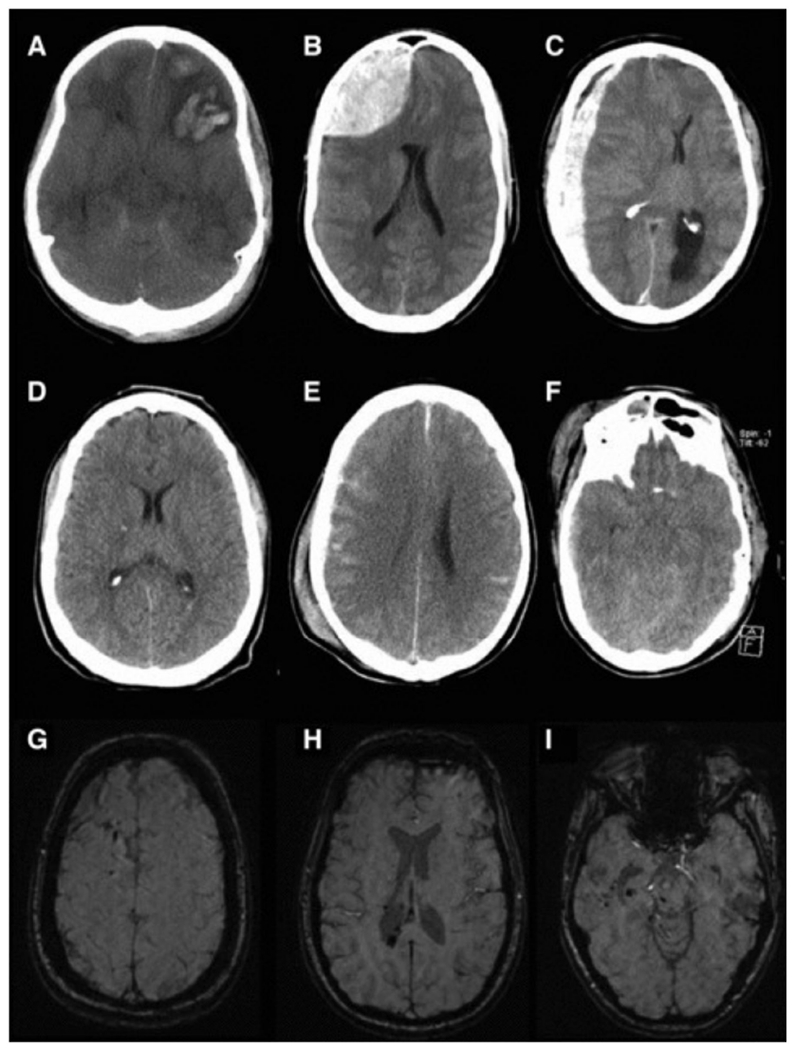
Examples of structural changes following focal and diffuse TBI represented by CT imaging (**A–F**) and MRI (**G–I**). (**A–C**) are of CT images following focal injuries, indicated by the presence of a focal contusion in (**A**), as well as hematomas in (**B**,**C**). Figures (**D–F**) are of CT images following diffuse injuries, indicated by hemorrhages in (**D**,**E**), and diffuse swelling in (**F**). Images (**G–I**) are of susceptibility weighted MRI images of one patient presenting with DAI indicated by hemorrhaging in different regions of the brain. Reproduced with permission from [27].

While focal injuries are particularly dangerous and concerning, special attention must be paid to diffuse injuries due to the underwhelming sense of urgency following trauma. Diffuse injuries describe an injury mechanism where rapid acceleration and deceleration results in semi-independent movements of brain structures due to the heterogeneous nature of tissue fixation with other structures and the skull, as well as tissue consistency [29]. This phenomenon is similar to the effect of whiplash following a traffic vehicle accident, where the brain’s inertia continues in the direction of the applied force, followed by a rapid deceleration against the inner wall of the skull. Directional movement influences the diffuse injury severity, as lateral movement tends to cause worse damage than sagittal movement [28]. This movement can result in vascular injury, brain swelling or edema, and most commonly, a diffuse axonal injury (DAI) [27,28]. DAI refers to the tearing of axons which, under normal conditions, would remain intact due to their high elasticity. However, when enough force is applied, the axons can tear or deform, resulting in permanent and irreversible damage to the fibers of neurons [27,28]. It is thought that this irreversible damage is caused by an initial swelling of the axon due to mitochondrial dysfunction leading to the collapse of the microtubular system throughout the cell, 6 to 12 h after the initial swelling [28]. However, there are other bodies of evidence that argue axonal swelling continues for years after the primary injury and could potentially contribute to increased disability in some patients [28]. Furthermore, Doppenberg et al. (2004) recommends excluding patients who are diagnosed with DAI from clinical trials until a proven therapy specifically for DAI is found in animal models [28]. Figure 1 provides both CT (A-F) and MRI (G-I) images of pathophysiological changes following both focal and diffuse TBI. This figure highlights the distinct structural differences between focal and diffuse injuries, which is important to keep in context when discussing the comparisons between animal models in the next section of this review.

The final mechanism of injury seen in TBI refers to non-impact injuries. Unlike focal injuries, non-impact TBI implies damage from injuries which did not result from direct penetrating or blunt force impact with the skull and is typically induced through alterations in pressure or acceleration/deceleration from the brain inside the skull. The associated pathophysiological consequences of non-impact injuries are unique due to the mechanism of impact, but share features observed in both focal and diffuse TBI. Additionally, clinical presentation of non-impact injuries is typically coupled with focal and diffuse injuries, leading to compounding effects on the pathological outcome. For example, those in military warfare can often be exposed to blast injuries, in which multiple mechanisms of injury are acting on the body. These elements include (1) primary blast injury: blast wave acting on the brain, (2) secondary blast injury: accelerated projectiles penetrating the skull, (3) tertiary blast injury: acceleration/deceleration effects acting on the body, and (4) quaternary blast injury: thermal and chemical injuries to the head following the initial explosion [30]. However, in this section of the review, we refer to the primary blast injury only. Blast waves result in accelerated air pressure which interacts with the head and body, creating acceleration or rotation of the head, and transfer of the kinetic energy from the blast through fluid circulating in the thorax [31]. Acceleration of fluid within the body results in increased intracranial pressure, which can result in BBB disruption, vasculature damage, edema, and hemorrhaging [30]. Cognitive deficits from blast injuries include headache, fatigue, problems with sleep and concentration, and even post-traumatic stress disorder, which is one of the behavioral aspects most relevant to members of the military. Additionally, road traffic incidents, as discussed briefly in the diffuse injury section, can produce rapid acceleration and deceleration of the brain inside the chamber of the skull, producing edema, vascular injury, and DAI [27,28].

While there are similarities between focal, diffuse, and non-impact injuries, each of these types of traumas produce unique pathological outcomes that are specific to the mechanism of injury delivered to the brain. Therefore, animal models must be developed with an in-depth knowledge of the mechanism of injury to enhance translation between the pathophysiological consequences seen following animal injury and clinical TBI. Through these animal models, researchers will be able to develop therapeutic options for alleviating the conditions presented within each type of TBI.

## 4. TBI Animal Models

Animal models are valuable tools used for providing an effective comparison to a variety of human conditions. Understanding the mechanism for the progression of various diseases allows researchers to develop treatment protocols which can be modified prior to human testing for optimal results. These models have been created for a multitude of ailments affecting the brain, including TBI [14]. TBI animal models have aided in the development of potential treatments for the reduction of oxidative stress, improving BBB permeability and other various biochemical impairments following TBI [8,14]. Several models have been developed, sectioned into three distinct categories as seen in clinical presentations of TBI: focal, diffuse, and non-impact injury [2,14]. Each model has distinct procedures and outcomes in the hopes of providing a sufficient translation to the variety of situations for which head trauma occurs in humans. Additionally, several of these models can be manipulated to alter the levels of injury severity, leading to a greater understanding of injury progression. Based on these experiments, comparisons are derived between the various degrees of human injury severity, which will ultimately lead to improvements in diagnostics and treatment protocols.

## 5. Focal TBI

### 5.1. Weight Drop

The weight drop model is one of the original methods used for assessing TBI and has several variations for modifying the overall design of the experiment [14,32–34]. These variations are effective in differentiating between the various mechanisms of injury caused by a force impacting the animal’s head. While each procedure varies slightly in design, each method follows the fundamental principles established by the weight drop method. Each of these models provide similar strengths in that the mechanism of injury is similar to human TBIs and each model has simplistic operations in comparison to some of the other injury methods discussed below. In these models, the animal’s head is placed directly under a free-falling weight, creating an impact between the animal and the load [14]. While the design of the model is consistent, manipulation of the mass and height of the free-falling weight allow for variation in injury severity, even within this own class of focal injury model [14]. For example, the kinetic energy created upon impact is related to the potential energy of the free-falling weight. Increasing the height of the weight or increasing the mass of the weight will both result in increased injury severity. There are several variations of weight drop models; however, the three focused on in this review are methods developed by Feeney, Shohami, and Marmarou—Marmarou is a unique diffuse weight drop model to be discussed later in this review.

### 5.2. Feeney’s Weight Drop Model

**Figure 2.**
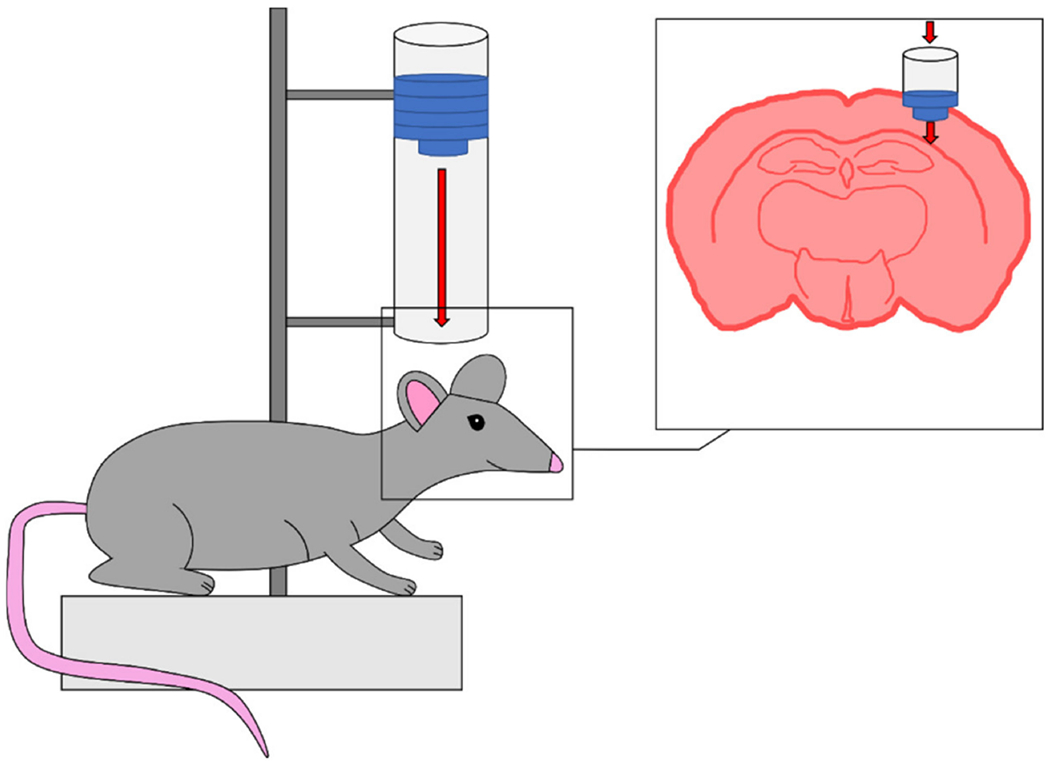
Illustration of Feeney’s weight drop model. In Feeney’s weight drop model, the weight is released inside of a secured column onto the intact dura of the animal’s brain. Figure inspired by Xiong, Y. et al. [14].

In Feeney’s weight drop model, an incision is made through the midline of the scalp to create clear accessibility to the skull below. A portion of the skull is removed through craniectomy to allow for a direct impact between the free-falling weight and the animal’s brain covered by the dura mater (Figure 2). The hole created from the removal of the skull is directly related to the diameter of the weight, reducing the risk of skull fracture from the weight colliding with the outer edges of the hole. For example, a cylindrical weight of 5 mm in diameter would require at least a 5 mm craniectomy. Craniectomies should not exceed the cranial defect size (5 mm for mice, 8 mm for rats) for each designated animal model to ensure adequate recovery of the calvaria, the cap of the skull [35,36]. After exposing the brain of the animal, Feeney’s weight drop design delivers the load directly onto the animal’s exposed, intact dura, producing a cortical contusion [33]. The initial impact produces hemorrhages in the white matter, directly under the impacted cortex, for several hours after injury, leading to the formation of a necrotic cavity at 24 h, expanding for two weeks [33]. Additionally, metabolic deficits were expressed as early as 2 days post-injury with analysis from magnetic resonance spectroscopy (MRS) indicating a reduction in N-acetyl aspartate, the most abundant molecule involved in CNS metabolism [37,38]. Recovery from functional behavioral deficits assessed by balance beam tests showed a dose-dependent relationship between trauma and injury severity, with deficits reported up to 90 days post-injury [14]. Regarding the strengths of this model, Feeney’s variation is simplistic in design and the immediate impact mimics the biomechanics of injury seen in moderate to severe human TBI, such as objects being accelerated against the skull. However, complications with the size and orientation of the weight in respect to the cranial defect can result in skull fractures, leading to challenges with reproducibility. Additionally, forces created for producing severe TBI (200–1000 g/cm) can result in higher rates of mortality, which reduces the reproducibility of the model [14,39].

### 5.3. Shohami’s Weight Drop Model

In Shohami’s weight drop model, the mechanism of impact is shifted to represent trauma in a closed head injury (CHI) experiment [14,32]. Prior to injury, an incision is made through the midline of the animal’s scalp to gain accessibility to the skull. However, unlike Feeney’s methods, this model does not require direct access to the brain through craniectomy, which can be beneficial for completely reducing the risk of damaging the dura prior to injury. Following the incision, animals are subjected to injuries produced with rounded free-falling rods [39]. Through alterations in the tip and the lack of craniectomy, this model represents a blunt impact to an unprotected skull differing from the penetrating mechanism of injury seen in Feeney’s model [14]. Additionally, some studies have installed the utilization of a rounded silicone tip for impact further reducing the chances of skull fracture, even with the exclusion of a craniectomy [39]. Physical impairments associated with this injury include BBB disruption, cerebral edema, and focal contusions, as well as cerebral hemorrhaging [14,32,39]. In mice, cerebral water content and BBB permeability increased in the ipsilateral region at 4 and 24 h, while alterations to the BBB remained for up to 30 days [32]. Additionally, cerebral edema, measured by linear specific gravity gradient columns, peaked in rats at 18 h following injury [40]. Biochemical changes associated with this CHI model have been studied extensively, indicating an elevated inflammatory response following impact [39,40]. Following injury, an increased production of prostaglandins in the ipsilateral region was shown from 18 h to 10 days post-injury, while immunohistochemical staining at 1 week post-TBI indicated an activation of microglia and astrocytes [41]. Behavioral deficits following injury were also evaluated using a Neurological Severity Score (NSS) assessment [14,32,40]. Scoring is calculated following the completion (or failure to complete) 10 assessments of physical, cognitive, and behavioral function [42]. Control animals, receiving no injury, are successful in completing each of the tasks and earn a score of 0, while animals presenting with severe deficits from injury earn scores of 10. Results from NSS indicate a strong correlation between behavioral deficits and injury severity, and elements of this assessment are discussed later in this review.

### 5.4. Fluid Percussion Injury

**Figure 3.**
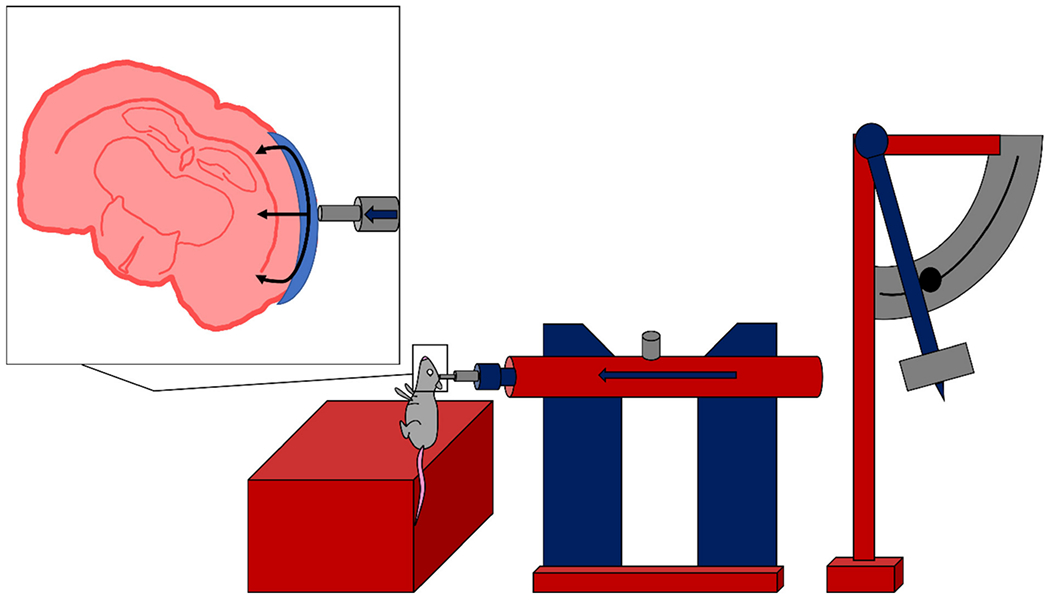
Illustration of the lateral FPI model. In the FPI model, impact results from the force of a pendulum striking a fluid reservoir, which generates a pulse of pressurized fluid to the undamaged dura, following craniectomy. Figure inspired by Xiong, Y. et al. [14].

Fluid percussion injury (FPI) models provide a mechanism of impact that has been shown to produce variable TBIs with a focal injury and characteristics of both focal and diffuse brain injuries [43–47]. Primary impact results from the force of a pendulum striking a fluid reservoir which generates a pulse of pressurized fluid to the undamaged dura following craniectomy (Figure 3). Surgical implantation of a Luer Lock tip over the region of interest following craniectomy is used to ensure a closed system between the fluid reservoir and the animal’s brain [46]. FPI models represent clinical injuries with no presence of skull fracture, which is typically seen in moderate to severe clinical TBI. Injury severity is determined by the intensity produced from the fluid pressure pulse, which can be altered by adjusting the angle of which the pendulum is released, similar to the adjusting the height of the weight in the weight drop models [14]. FPI models also tend to have less control over the injury as the pendulum height is the only variable when using FPI models. However, the FPI method has been replicated in a variety of animal models, including cats, rabbits, rats, sheep, mice, and swine, and impacts have been characterized for injuries located at either the midline or lateral portion of the animal’s brain depending on the location of the craniectomy [14,43,44]. Midline FPI places the craniectomy at the center of the sagittal suture while parasagittal and lateral models place the center of the craniectomy at <3.5 mm or >3.5 mm lateral from the midline, respectively [14]. While lateral FPI localizes the pressure pulse to a specific region of interest (focal), midline FPI has been suggested to mimic characteristics of a diffuse injury due to the presentation of mild behavioral deficits and lack of gross pathological changes to the structures of the brain [46].

### 5.5. Lateral Fluid Percussion Injury

**Figure 4.**
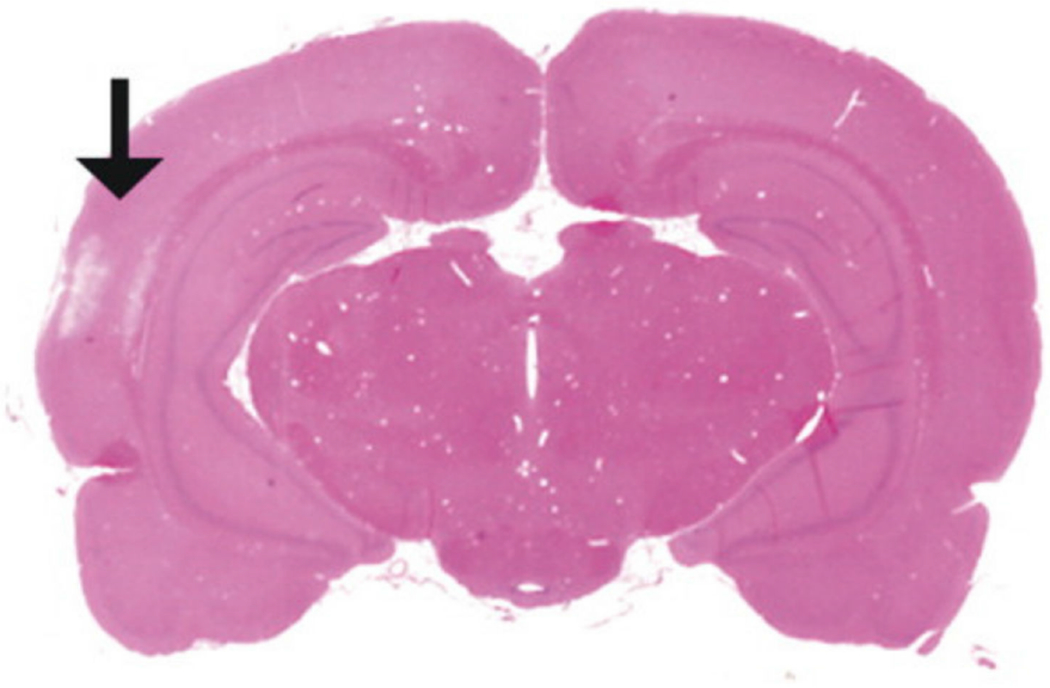
Hematoxylin and eosin (H&E) staining of a coronal brain section at 7 days post-LFPI. Black arrow indicates gross pathological changes at the site of injury. Reprinted with permission from [49]. Copyright 2006 Society for Neuroscience.

Lateral FPI models are classified into mild (26–32 psi), moderate (35–41 psi), and severe (>41 psi) injuries based on the pressure pulse of the fluid [48]. For lateral FPI, the center of the craniectomy is positioned <3.5 mm or >3.5 mm laterally from the midline for parasagittal and lateral injuries, respectively [14,45]. Due to this lateral placement, pathological changes are not typically seen in the contralateral hemisphere of the brain (Figure 4) [49]. Additionally, lateral FPI models do not produce skull fractures, which are characteristically seen in clinical moderate to severe TBI [45]. However, features associated with lateral FPI include edema, intracranial hemorrhages, and progressive damage to gray matter, which is consistent with the pathophysiology of TBI [45]. Lateral FPI in rats results in focal cortical contusion with diffuse subcortical axonal injury and intra-parenchymal hemorrhaging [50]. Evaluation by Nissl staining indicated neuronal damage in the ipsilateral cortex, hippocampus, and thalamus as early as 12 h post-FPI [50]. Additionally, acute changes in the ipsilateral cortex following moderate parasagittal FPI progress into the formation of a cavity, which will continue to expand up to one year post-injury [51].

### 5.6. Penetrating Ballistic-Like Brain Injury

**Figure 5.**
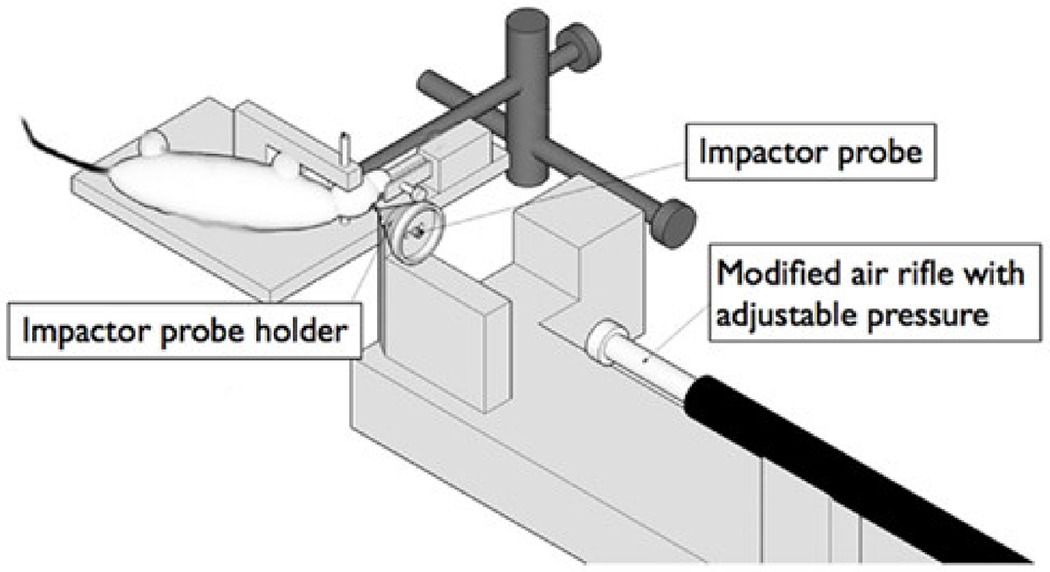
Illustration of the apparatus used in the PBBI model. In the PBBI model, impact is generated from the acceleration of a projectile into an impactor probe creating a temporary brain cavity in the animal model. Reproduced with permission from [56].

The penetrating ballistic-like brain injury (PBBI) model represents an injury consistent with severe TBI with a mechanism of injury similar to a gunshot wound [52–55]. PBBI models produce an impact through the acceleration of a high-energy projectile into an impactor probe placed inside a cranial window, creating a temporary brain cavity in the animal model (Figure 5) [56]. Following craniectomy, the impactor probe is inserted through the cranial window, while a water-filled balloon is inflated/deflated to generate a temporary cavity in the cerebrum. The impactor probe is typically cone shaped, mimicking the injury created following a gunshot wound and creating a specific translation to the biomechanics of human injuries. Acute changes following injury have shown increased intracerebral hemorrhaging, with maximum volumetric size occurring at 6 h post-TBI [53]. Injury progression leads to the development of a lesion of degenerate neurons at 24 h post-TBI [53]. Lesions resulting from PBBI have shown to be lined with neutrophils and macrophages at 24 and 72 h post-injury, respectively. Features associated with the acute phase of injury also include degeneration of white matter, edema, and gliosis, in addition to the tissue destruction and cavity formation identified previously [54]. The PBBI model provides a unique translation to severe penetrating injuries; however, due to the high-energy impact created in this design, mortality rates of the animals are a concern if the velocity of the impactor is not adjusted to reduce overall brain disruption.

### 5.7. Controlled Cortical Impact

**Figure 6.**
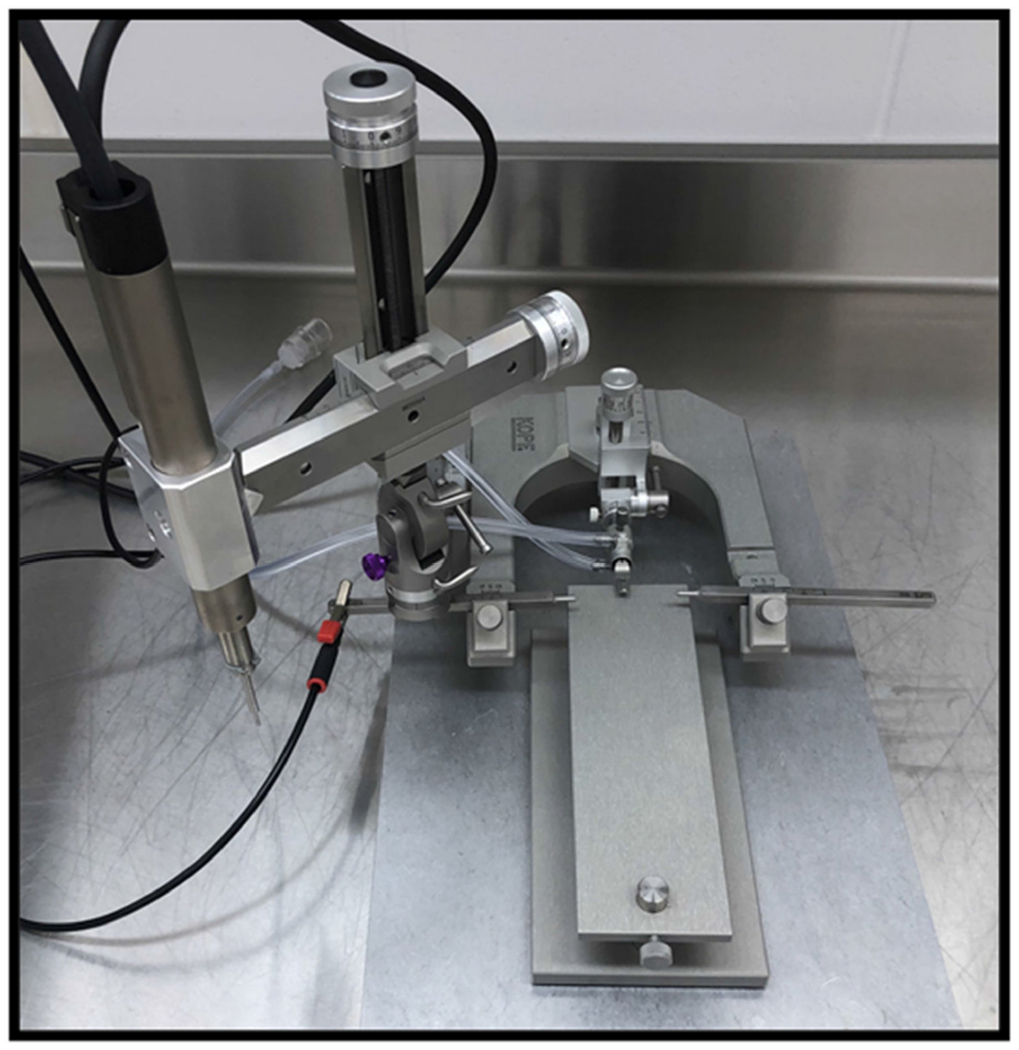
Example of an electromagnetic CCI system with stereotaxic frame for stabilizing mice.

**Figure 7.**
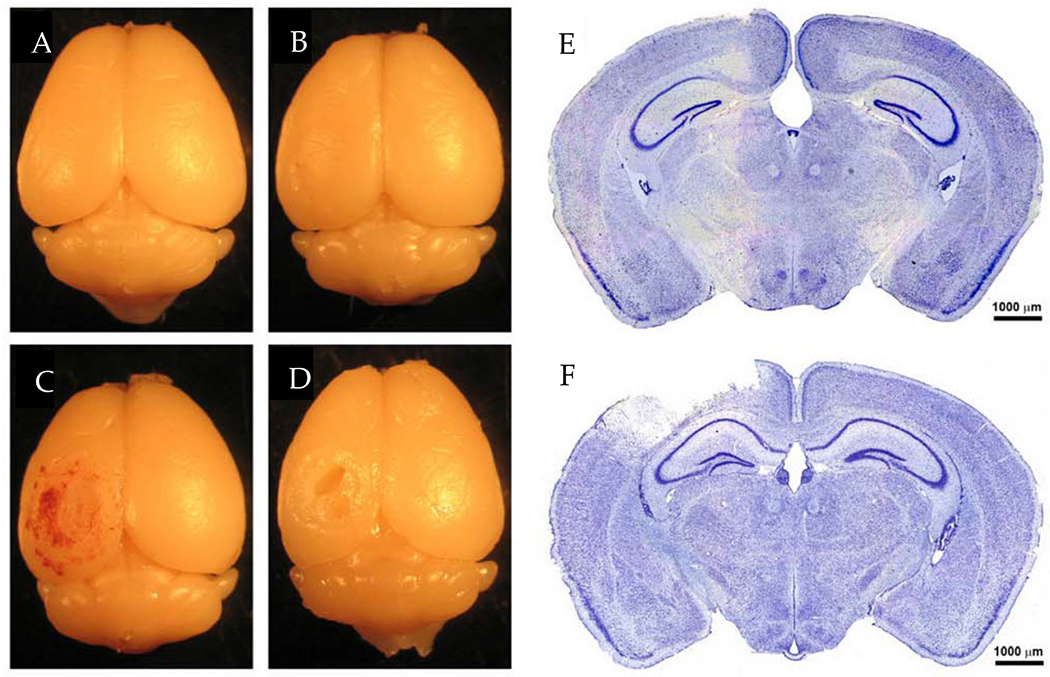
Brains collected from experimentation in the CCI model [60]. (**A**) 10-week-old mouse, (**B**) sham (craniectomy only), (**C**) 24-h post-moderate TBI, (**D**) 6-week post-moderate TBI, (**E**) Nissl staining of sham, (**F**) Nissl staining of moderate TBI. Adapted with permission from [60]. Copyright 2014 MyJoVe Corporation.

The controlled cortical impact (CCI) model is currently one of the most used and well-characterized models of TBI due to the model’s reproducibility and specificity regarding mechanical parameters [57–60]. CCI models use a pneumatic or electromagnetic (Figure 6) impact system to deliver a rigid impactor onto the exposed dura of the animal following craniectomy [58]. Originally developed in ferrets, the CCI model has been adapted for a variety of species, including mice, rats, swine, and monkeys [14,59]. Features of injury include subdural hematoma, subarachnoid hemorrhage, and axonal injury, in addition to cortical contusions and cortical tissue loss, which have been shown in clinical presentations of TBI [58–60]. Primary advantages of using CCI models include precise automated control over a variety of factors such as impactor diameter, velocity, depth, and dwell time of impact [60]. Previous literature has identified the appropriate depths for inducing mild, moderate, and severe TBIs as 0.0–0.2 mm, 0.5–1.0 mm, and 1.2–2.0 mm, respectively [60]. Figure 7 shows whole brain images and histological images of coronal brain slices following a moderate TBI with a velocity of 3.0 m/s, tip diameter of 3 mm, and depth of 1 mm. Images from 24 h and 6 weeks following moderate injury show cortical tissue loss in the ipsilateral hemisphere (Figure 7C,D), in addition to the loss of Nissl-stained neurons (Figure 7F) [60].

## 6. Diffuse TBI

### 6.1. Marmarou Weight Drop Model

**Figure 8.**
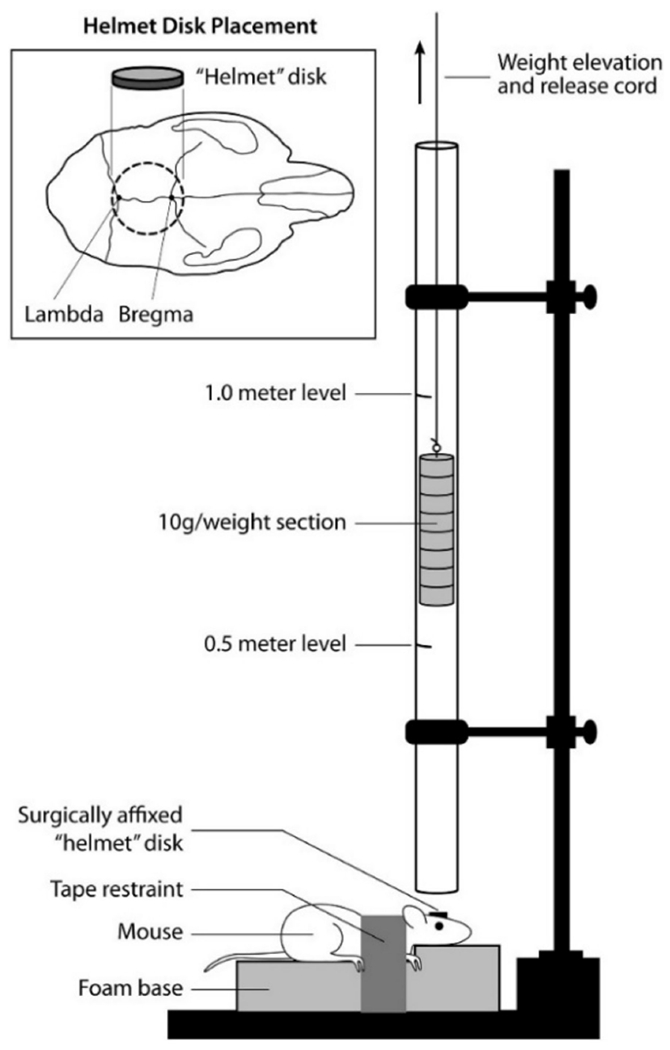
Illustration of a modified grade 1A Marmarou weight drop model. In the Marmarou weight drop model, impact is delivered through a free-falling weight colliding with a helmet secured to the animal’s head. The animal is placed onto a foam pad to decelerate impact and reduce the risk of contrecoup injuries. Reproduced with permission from [62]. Copyright 2016 Elsevier.

The Marmarou weight drop model has a distinct experimental design that mimics human diffuse TBI through the utilization of additional equipment that impacts a greater surface area of the skull and diffuses the primary injury throughout the brain [34,61]. Following a midline incision into the animal’s scalp, a stainless steel disc is attached to the skull with an adhesive glue between the lambda and bregma [34,61]. This disc is used to prevent skull fractures upon impact from the free-falling weight, which is more frequent in the focal injury weight drop models. Additionally, the animal is placed onto a foam bed to reduce the deceleration of the animal’s head following impact (Figure 8) [62]. This reduction in deceleration mitigates the risk of producing contrecoup injuries opposite the impact [14]. In a study conducted on rats in 1994, animals were impacted with a weight of 450 g from heights of 1 or 2 m [34]. Animals injured from 1 m had no mortalities, while heights from 2 m resulted in a 59% mortality rate [34]. However, groups receiving intervention in the form mechanical ventilation did not suffer mortality for either height [34]. Both heights produced diffuse brain injuries with no presence of focal lesions, while petechial hemorrhaging was associated with injuries produced from the 2 m height [34]. Neuronal injury was noticed in both ipsilateral and contralateral cortices, in addition to DAI present in the corpus callosum, long tracts in the brain stem, and to the cerebral and cerebellar peduncles [34]. Due to the presentation of DAI following impact, Marmarou’s model has been well characterized in literature; however, it has been associated with a high mortality rate due to respiratory depression without mechanical ventilation following injury.

### 6.2. Modified Marmarou Weight Drop Model

**Figure 9.**
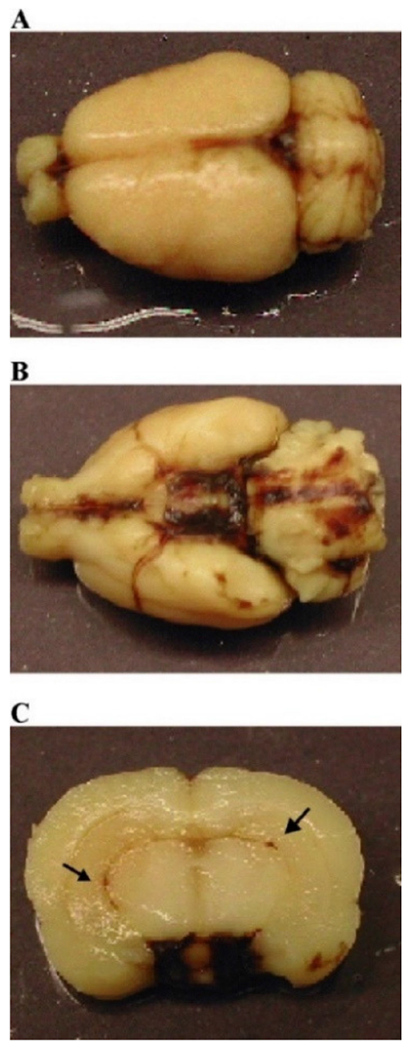
Brain from a moderate diffuse injury model 24 h following impact. (**A**) Superior surface, (**B**) Jnferior surface, (**C**) Coronal view. Black arrows indicate presence of subarachnoid and intraventricular hemorrhages. Reprinted with permission from [63]. Copyright 2004 Elsevier.

While Marmarou’s weight drop model has shown to be successful in producing features of diffuse injuries such as DAI, limitations in reproducibility have led researchers to explore alternatives to the original methods established in 1994. The diffuse injury model developed by Cernak et al. in 2004 incorporates a variety of factors from the Marmarou weight drop model and the CCI model to develop a reproducible diffuse moderate injury [63]. Following a midline incision through the scalp, a steel disc (10 mm diameter, 3 mm thickness) is cemented to the animal’s skull using a polyacrylamide adhesive [63]. The impactor tip uses the same steel disc as the one attached to the animal’s head, so that there is no impact to the unprotected skull, minimizing the risk of fractures [63]. Lastly, the animal’s head is supported by a molded, gel-filled base, similar to the foam base in Marmarou’s model [34,63]. This base is used to decelerate the animal’s head upon impact to prevent any injuries produced between the animal and the hard surface below. The impact is produced by an air-driven high-velocity impactor, similar to the pneumatic system used in CCI with a velocity of 3.25 m/s [58–60]. Additionally, the depth of impact was 18 mm for this moderate TBI, with a mortality rate of 26%. However, a range of depths from 16 mm to 20 mm was tested, with depths of 19 and 20 mm representing severe TBI at 56% and 90% mortality rates, respectively. This model showed increased edema and BBB permeability as early as 20 min following moderate injury. Additionally, measurements in arterial blood pressure increased immediately following injury and declined, reaching a minimum at 1 min post-injury, which was shown previously in Marmarou’s weight drop model [34]. Features of this diffuse model include no focal lesions or contusions, with presence of subarachnoid and intraventricular hemorrhages (Figure 9C, black arrows) [63]. Overall, this model provides unique advantages for producing DAI with enhanced reproducibility and reduced mortality rate through the incorporation of an air-driven impactor capable of making precise, automated adjustments to parameters such as speed and depth.

### 6.3. Modified Controlled Cortical Impact

**Figure 10.**
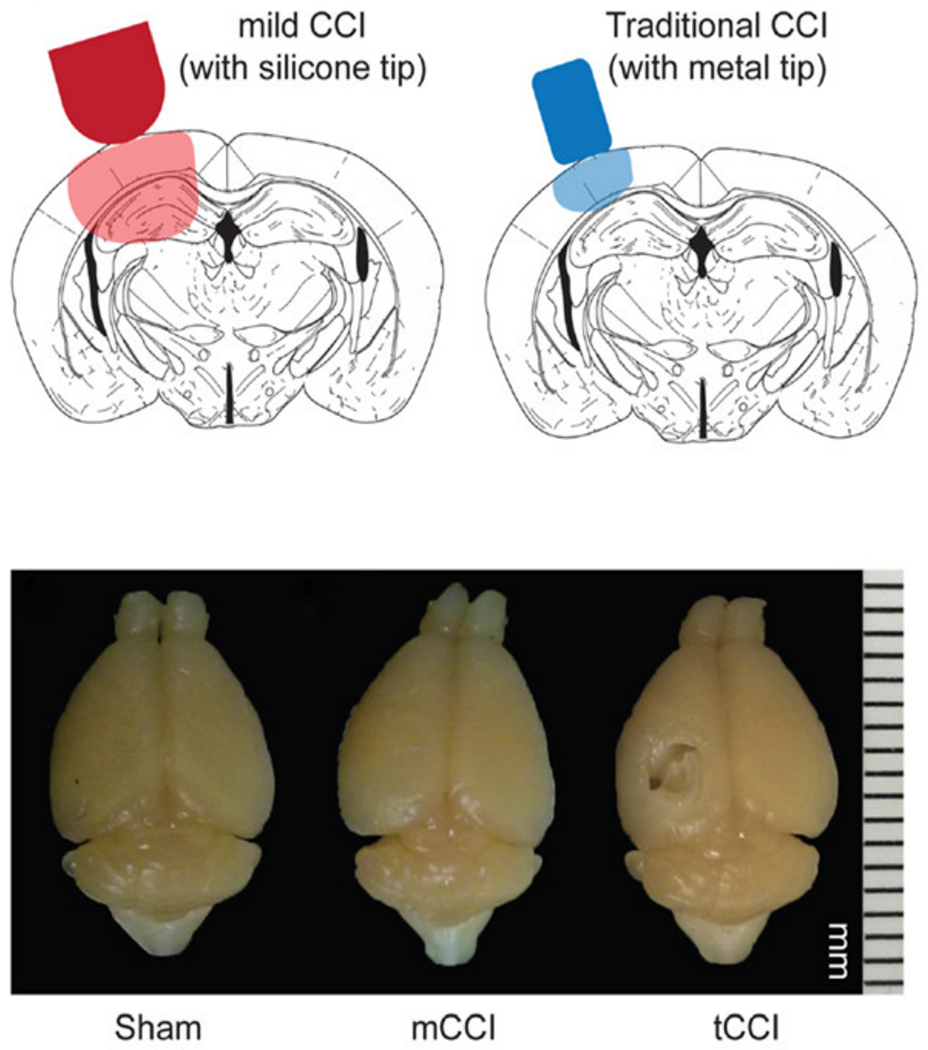
Modified controlled cortical impact model. **Top**: Comparison between the impactor tip size and region of injury between mild and traditional CCI [64]. **Bottom**: Brains 8 days post-injury showing comparisons between sham, mild CCI (mCCI), and traditional CCI (tCCI). Reproduced with permission from [64].

For the investigation into the biomechanics involved in mild TBI, in 2014, Meaney et al. introduced a modified CCI model through adjustments to the mechanical parameters discussed previously, in addition to the material and size of the impactor tip. This modified CCI model uses similar methodology and equipment as the previously discussed CCI model, but with a much lower impact velocity of 0.43 m/s and a larger impact depth of 2.1 mm. The material and size of the impactor tip was adjusted to produce a diffuse, mild injury. In this study, the impactor tip (4.0 mm diameter) was manufactured from Sylgard-184 to produce a soft silicone tip capable of producing a diffuse injury across a greater surface area of the brain [64]. Figure 10 (top) illustrates the comparison in tip size and region of injury between the mild CCI (mCCI) impactor tip developed in this study and the traditional CCI impactor (tCCI) tip made of metal, typically stainless steel [64]. Features of this model include subcortical axonal injury, with no presence of visible lesions or hemorrhaging (Figure 10, bottom). An additional point of consideration highlighted in this figure is the lack of cortical lesion represented in both the sham and mCCI brain images. Several reports have discussed the impact of craniectomies in elucidating changes in inflammatory and behavior responses. Therefore, the incorporation of a sham model is crucial in separating the effects from injury and surgical perturbation of the skull. This injury design further illustrates the variation established with the use of CCI methods. While this method requires further standardization, the variation in impactor tip hardness provides the possibility for additional studies with ranging injury outcomes.

### 6.4. Repeated Mild TBI

The pathological and cognitive outcomes following repeated mild head impact, including concussions or sports-related head trauma, have been recently addressed with the introduction of CTE [5,65]. CTE was first reported in a retired National Football League player with neurological impairment and symptoms of Parkinson’s disease [65]. Due to the relationship between repeated mild TBI and neurodegenerative diseases [5,65,66], researchers have also been interested in the pathological changes corresponding to increased frequency of mild TBI in animal models [66]. Several experiments have been designed for determining how the frequency of TBI induces acute as well as chronic changes in animal models. As described in a review by Hiskens et al., experimental studies have administered injuries using modified weight drop, lateral impact, and modified CCI methods [66]. Injury frequency ranged from 1 to 42 impacts, with intervals ranging from 3 min to 1 month [66]. These experimental studies into the pathological and neurological outcomes following repeated mild TBI will continue to build upon our current understanding between the relationship of TBI and neurodegenerative diseases.

## 7. Non-Impact TBI

Non-impact TBI animal models provide an alternative mechanism for clinical presentations of injury that are not produced directly from mechanical impact. The previous injury models have all been representative of a human TBI developed from an initial mechanical force delivered to the head. However, there are additional circumstances that can result in the production of a TBI without direct impact. Two examples of non-impact TBI discussed below result from rotational acceleration and blast waves. These animal models are specific in their ability to represent head trauma in humans.

### 7.1. Closed-Head Impact Model of Engineered Rotational Acceleration (CHIMERA)

**Figure 11.**
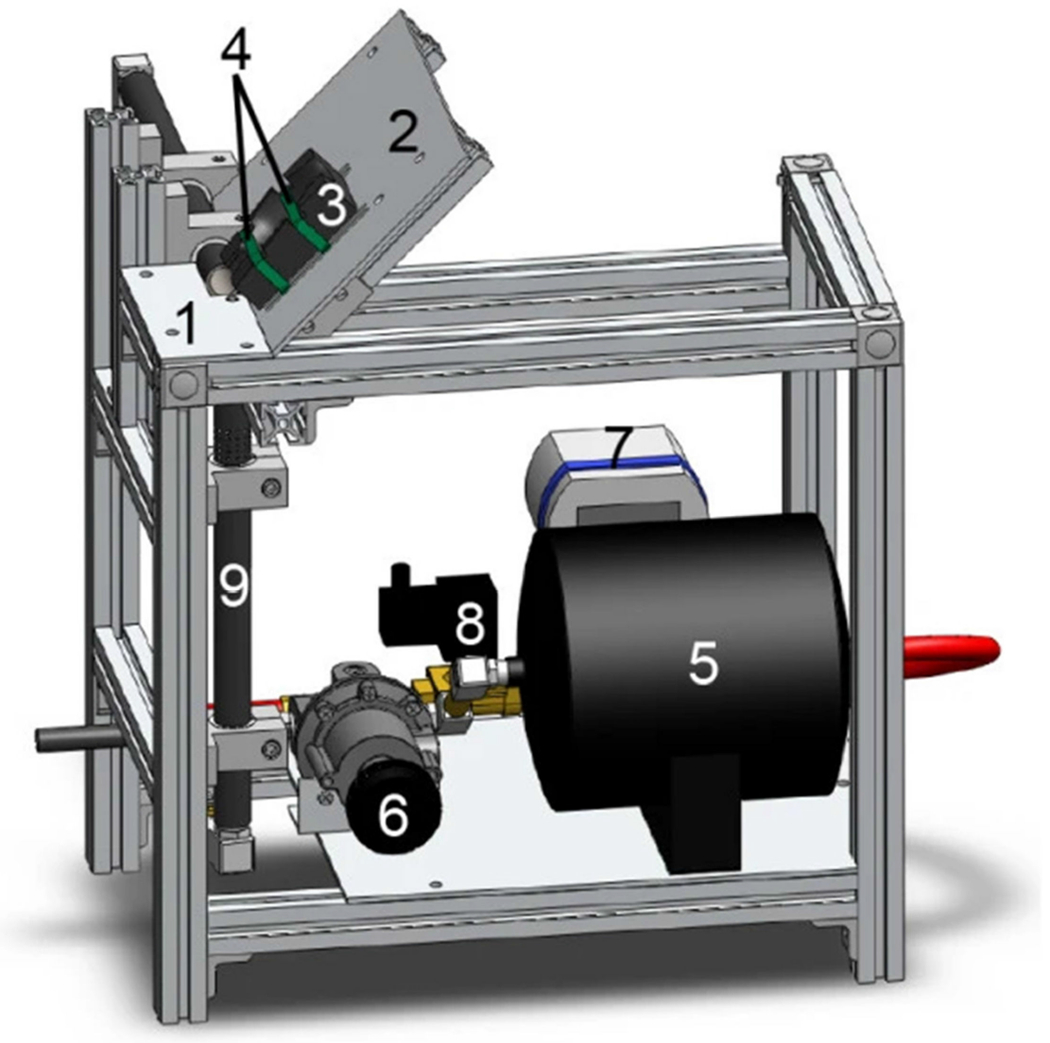
Illustration of the CHIMERA device. Portions of the device are labeled with numbers including: 1. head plate, 2. body plate, 3. animal bed, 4. Velcro straps, 5. air tank, 6. air pressure regulator, 8. two-way solenoid valve, 9. vertical piston barrel. Reproduced with permission from [4].

**Figure 12.**
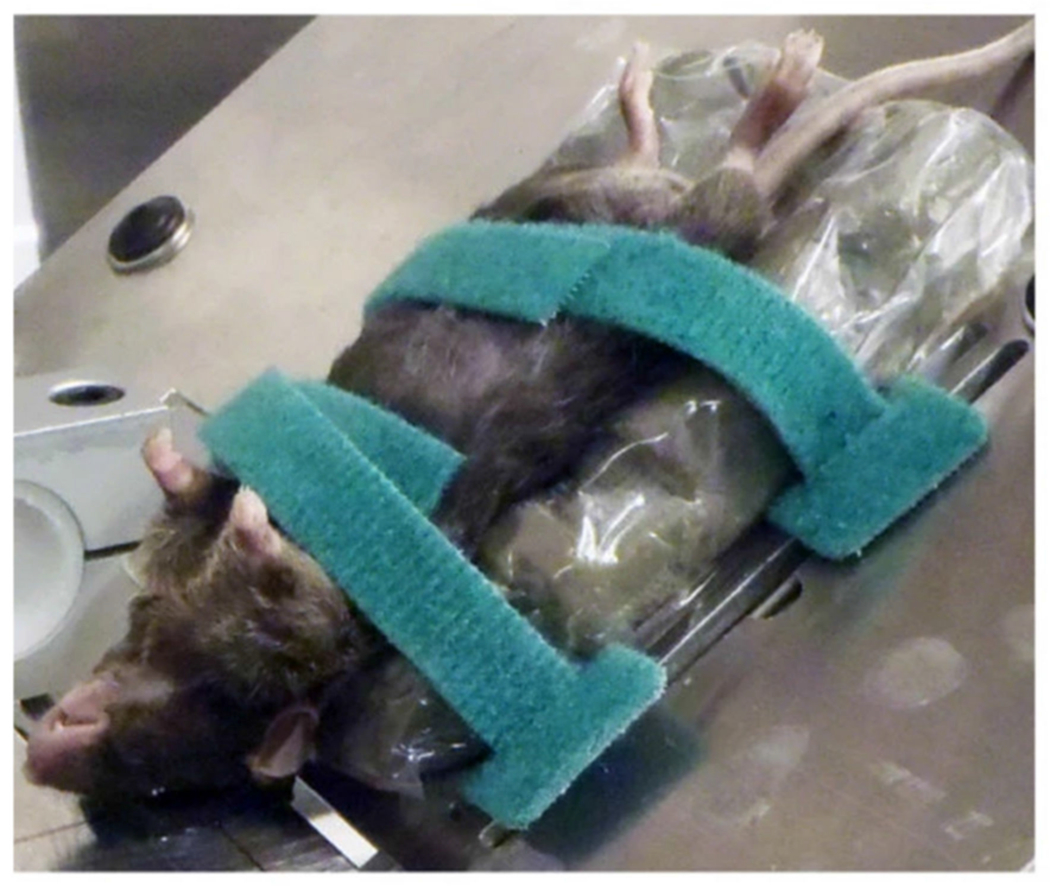
Positioning of animal prior to the induction of injury, secured firmly with Velcro straps allowing free rotation of the head and neck. Reproduced with permission from [4].

The CHIMERA model was designed to produce a repeatable CHI in rodents through frontal rotational acceleration of the head without the need for surgical intervention [4]. In 2014, Wellington et al. studied the relationship between biomechanical movement of the brain and pathological characteristics observed in clinical TBI [4]. Illustration of the components involved in the CHIMERA device is provided in Figure 11 [4]. In this study, no craniectomy or surgical intervention was required prior to injury, as the mouse is attached directly to a body plate using Velcro straps with no restriction to the mobility of the head (Figure 12) [4]. Once the animal has been secured, pressurized air drives a piston upward to produce an impact to a plate that the animal’s head is resting on with a kinetic energy of 0.5 J [4]. However, the desired impact velocity and energy can be calculated by making incremental changes in pressure used for firing the steel piston. The following impact to the plate produces a frontal rotation of the animal’s neck and head, similar to the effects of whiplash following a motor vehicle collision. In this study, high-speed videography was used to analyze the kinematics following two repeated TBIs (rTBI) with 24 h separating each injury. Elements of rotational acceleration were analyzed, including head trajectory and displacement, in addition to linear and angular velocity and acceleration of the head. Additionally, immunohistochemical analysis at 2, 7, and 14 days post-rTBI showed microglial activation through the white matter tracts of the brain, including corpus callosum and optic tracts. This novel experimental model was also able to replicate DAI following rTBI, without the need for surgical intervention or direct impact between a weight and the skull. Additional studies have been conducted using the CHIMERA model for both moderate injury and for exploring the pathological changes in transgenic mice for Alzheimer’s research [67,68].

### 7.2. Blast Injury Model

**Figure 13.**
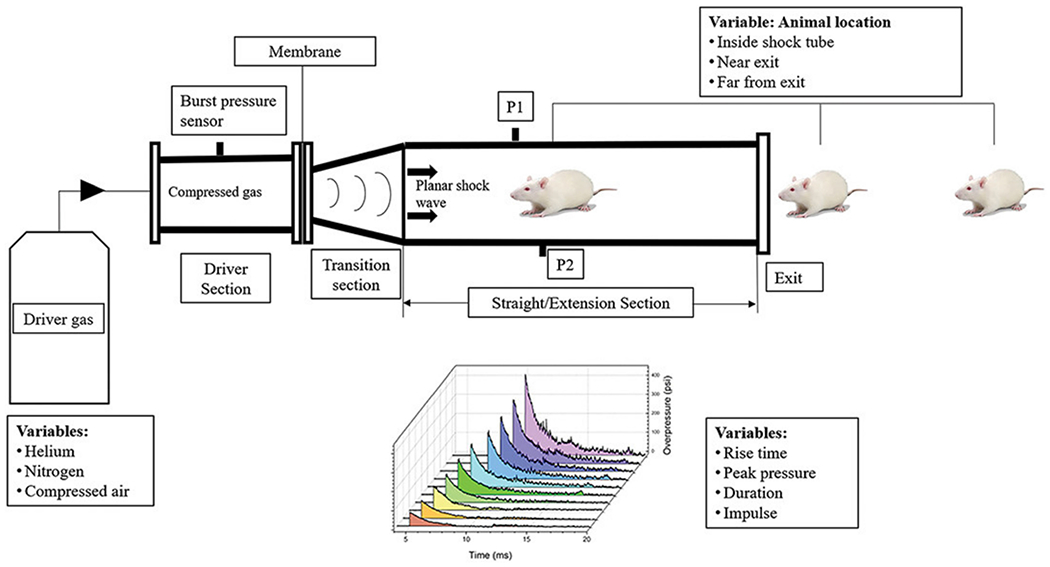
Illustration of the experimental design of a blast injury model, including alterations made from individual studies. The blast injury model produces energy waves by releasing compressed gas through a tube to simulate blast effects in an animal without the need to expose the skull. Reproduced with permission from [69].

Blast injury models have been extensively characterized for understanding the mechanism of injury relevant to military combat. While clinical presentations of blast-induced TBI typically includes multiple levels of injury [30], the pathophysiology following primary blast injury requires its own individual model and experimentation. These models produce energy waves by releasing compressed gas through a tube to simulate blast effects in an animal without the need to expose the skull (Figure 13) [3,69]. The animal is placed inside of or directly near the tube, and the detonation delivered from the blast produces waves of energy that result in the injury [3,14]. Features of blast trauma in rats include brain contusion, laceration, hematoma, as well as axonal injury in the cerebellum and brainstem [3,70]. Additionally, following a single blast exposure of 35 psi, axonal degeneration was present at 24 h, 72 h, and 2 weeks post-blast injury. Studies have also been developed to understand the neurological effects of the animals, in addition to the effect of Kevlar vests and body shielding in protecting the thoracic portion of the animal’s body [3,70]. In one study, additional body shielding resulted in decreased mortality and improvement in fiber degeneration in the brains of rats following a 126 kPa air blast. Lastly, blast-related mild TBI has also been correlated with the development of PTSD [30], leading researchers to explore the cognitive deficits following blast injuries. Ultimately, the development of this model provides a unique tool for understanding the progression of neurological conditions experienced by non-impact blasts.

## 8. Behavioral Analysis

Animal behavior is a common method of determining deficits post-TBI when using the above-described animal models. For these analyses to be sufficient in determining neuroprotective capabilities of nanotheranostics, it is important to consult with a behavioral specialist or acquire the proper training needed to carry out these procedures with confidence. Additionally, it is essential to determine which results will be measured prior to the beginning of an experiment to avoid p-hacking or misinterpreted results. The model used for testing is crucial for behavior as severity, phase of secondary injury, number of injuries, area of impact, and type of injury have been shown to show differences in behavior post-TBI [69,71–73]. Thus, anyone looking to utilize behavioral analyses must be aware of any potentially confounding issues that may result from motor deficits, visual impairment, animal strain, sex differences, or other issues that may arise during testing. While the assessments of tasks below are useful for determining which tests can contribute to the study of TBI therapies, the explanations of results described in Table 2 are accurate only when no confounding factors are present. With that stated, there are various forms of behavioral analyses one could benefit from using that are categorized into four groups of tasks: spatial learning and memory, nonspatial learning and memory, emotional, and motor coordination.

## 9. Spatial Learning and Memory Tasks

Spatial learning and memory are governed by the ability to navigate with two forms, allocentric and egocentric navigation. Allocentric navigation is generally described as using distal spatial cues to guide the direction of movement while egocentric navigation relies more heavily on internal cues such as remembered sequence, speed, the direction of movement, and utilizing closer cues referred to as “signposts”. Important in the discussion of egocentric versus allocentric navigation is distinguishing between “signposts” and “landmarks”. While they provide information for egocentric and allocentric navigation, respectively, signposts do not provide any relational information. Signposts simply convey where to change direction and do not aid in understanding where one is in comparison to other signposts. In contrast, landmarks do not inherently tell you where to change direction, but can provide key information regarding one’s placement in relation to other landmarks [74]. To better understand, think of signposts as a particular intersection where you know to turn right to reach your location. Inversely, one could also use the landmark of the street sign and the knowledge of the direction they are approaching from to know to turn right in that situation. While these can sometimes result in the same or similar choices, such as in this example, that is not always the case. For the sake of consistency, egocentric navigation will be covered as a form of nonspatial navigation; therefore, our focus in this section is the allocentric aspects of each of these paradigms despite the interconnected nature of the two forms of navigation. In order to simplify this review, allocentric navigation will be the only form discussed within this section as it focuses on hippocampal activity even though both allocentric (spatial) and egocentric (nonspatial) navigation systems have an overlap in healthy brains [74].

### 9.1. Morris Water Maze and Barnes Maze

**Figure 14.**
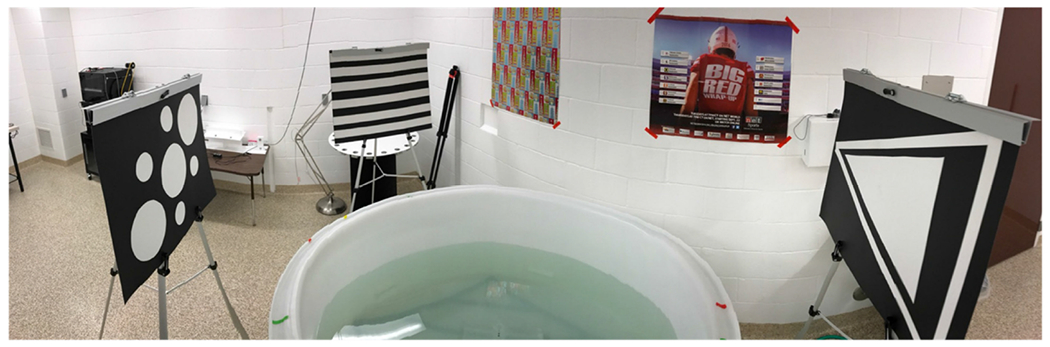
Example of a MWM set up, including spatial cues.

**Figure 15.**
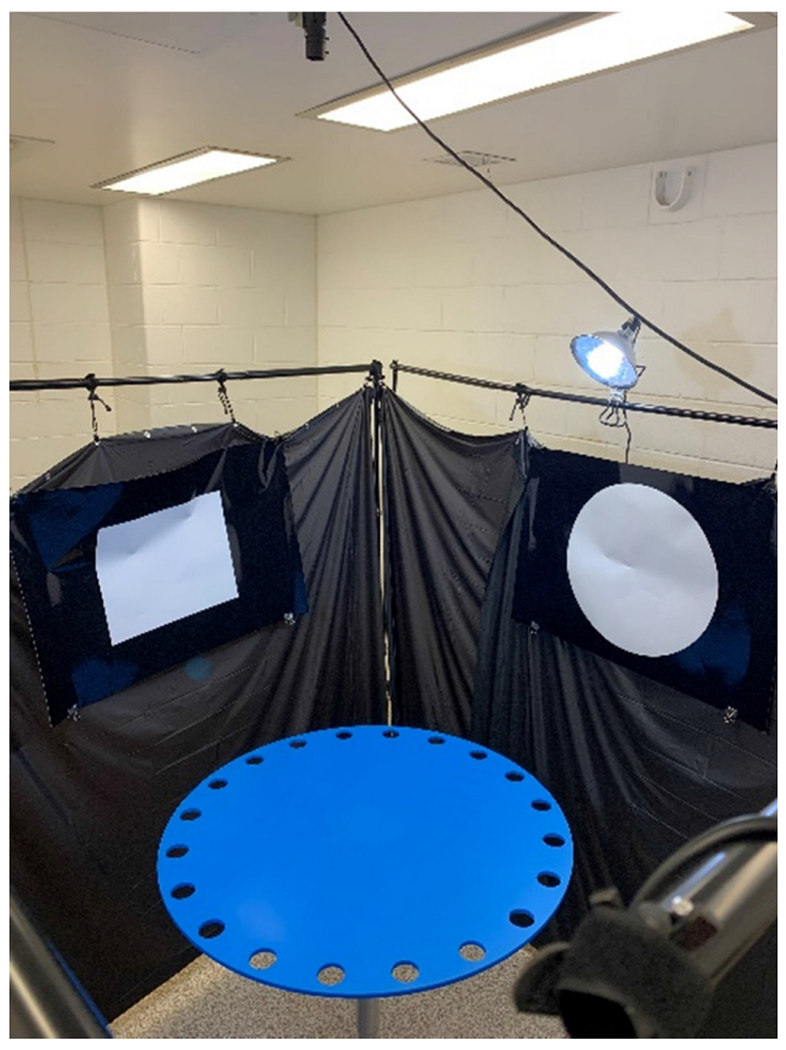
Example of a BM set up with spatial cues and overhead lighting.

Two tests often utilized when determining behavioral deficits in rodent models, which are the most utilized in TBI research, are the Morris water maze (MWM) (Figure 14) and the Barnes maze (BM) (Figure 15). Both tasks aim to determine a test subject’s spatial learning and memory skills without a restriction to movement. Each test has similar features, such as extra-maze visual cues facing toward the maze in the north, south, east, and west directions. It is worth noting that these are arbitrary distinctions and not related to compass directions. The goal of these tests is to find an escape area, particularly a hidden platform in the MWM and an escape box in the BM, that remains static throughout each week of training, with the start location randomized to ensure allocentric navigation. Additionally, both tests can utilize a reversal trial where the escape area is located opposite of its placement the week prior to test the ability to relearn spatial navigation. Standard protocol usually has these escape areas in the southeast quadrant for the first week and the northwest quadrant in the reversal week [75,76].

**Figure 16.**
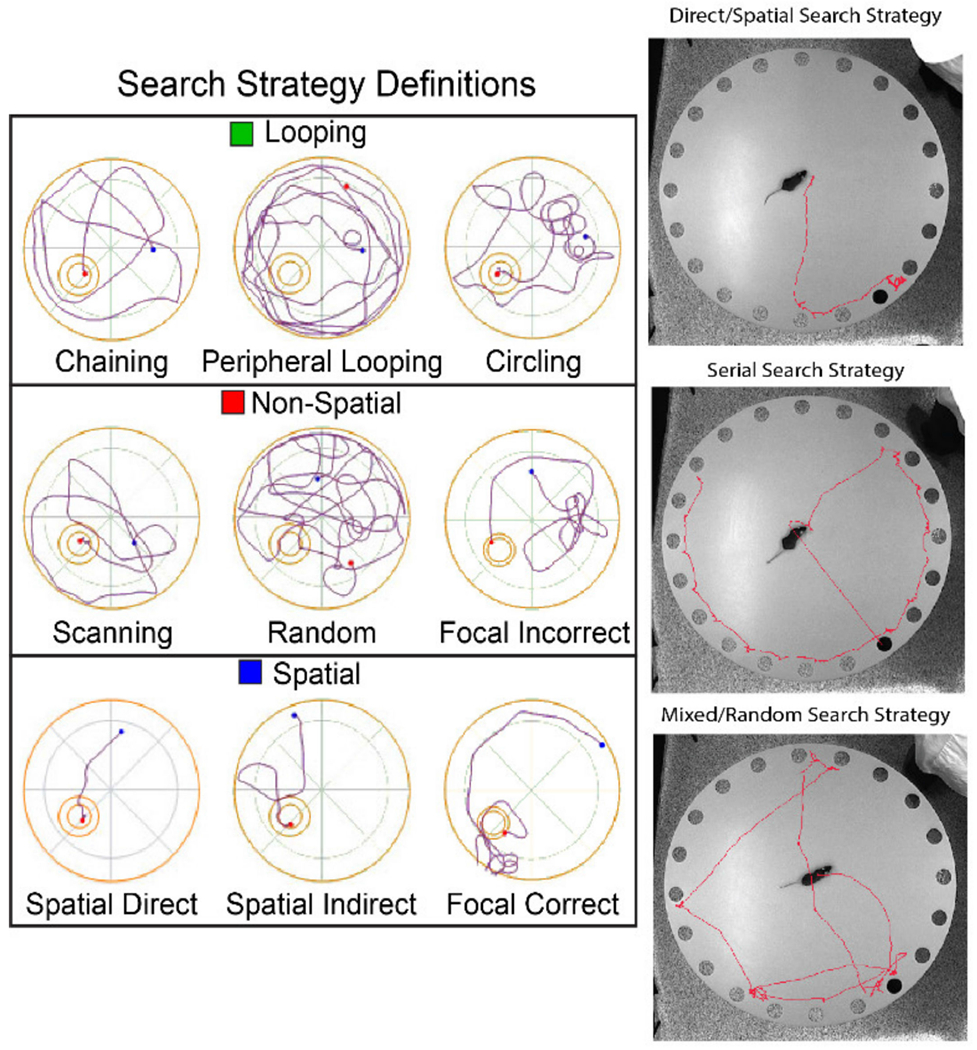
On the left, MWM search strategies are defined into their three primary categories and three subcategories. On the right are the BM search strategy categories. Reprinted with permission from [81]. Copyright 2021 Elsevier.

Despite many similarities, there are also various differences between the two maze styles. The MWM differs from the BM as it uses a negative environmental factor, water immersion, to promote learning [75]. Water immersion causes high stress and tends to result in an increase in corticosterone levels in plasma when compared with the BM [77]. While this may be the biggest difference, the MWM also uses a different search strategy analysis due to its vastly different methodology. These search strategies can show if the animal is learning through visual cues, geometric information of the maze, or random behaviors [78]. When quantifying search strategy data for the MWM, three groups of strategies, each with three subgroups, are determined: spatial, non-spatial, and repetitive looping strategies. The subgroups are as follows: for spatial strategies, there are spatial direct, spatial indirect, and focal correct strategies; for non-spatial strategies, there are scanning, random, and focal incorrect; for repetitive looping strategies, there are chaining, peripheral looping, and circling. These spatial strategies can show differences in learning between the spatial and non-spatial groups versus the repetitive looping groups due to the association between the hippocampus and memory of spatial landmarks in relation to the subject’s goal [78]. In comparison, the BM has a much more simplified search strategy analysis which consists of direct, serial, and mixed (or random) strategies [75]. Direct strategies are defined as a direct movement toward the target hole or to the holes adjacent to the target. Serial strategies are defined as strategies where the animal first visits a hole non-adjacent to the target and follows in a clockwise or counterclockwise rotation to each hole until the target is found. Mixed, or random, strategies are defined as a series of hole searches separated by movement across the center of the maze or a generally unorganized search. Figure 16 exemplifies each set of search strategies using previously published examples and new search strategy examples. Other useful data to be gathered from these tasks are the primary escape latency, where the animal first looks inside of the target hole, and the number of primary errors, referring to the number of times the animal attempted to escape through a non-target hole [75].

Both the MWM and BM produce a wide variety of data able to be derived from each experiment. While all data are useful in specific contexts, certain measurements, such as the latency to escape, path length, and cumulative distance from platform for the MWM [76], and the primary latency, primary errors, and total path length for the BM [75], are more useful for TBI testing, while some are just generally more useful and highly utilized in other research contexts. The various changes between injured animal data with the amount of available data is covered in Table 2, which also provides the expectations one should have regarding how injured animals compare to controls, as well as the reasoning behind each datum.

Due to the widespread use of these mazes in preclinical testing, virtual reality (VR) forms of multiple spatial paradigms have been created to measure cognitive deficits in a clinical setting while remaining both ethical and practical. VR has created a unique opportunity for clinical researchers to draw direct correlations between preclinical and clinical testing by placing patients in a virtual environment similar to that experienced by preclinical rodent models. The MWM VR experience has been highly explored [79]; however, no BM paradigm has yet to be created. Despite this and a lack of endogenous stress in VR, much of the data gathered using the VR MWM may be somewhat translational and help to connect clinical success with preclinical testing. Additionally, VR MWM’s have shown a connection between VR testing and rodent testing through the performance relying on hippocampal and medial temporal lobe integrity, among other similarities [79,80]. These two tests have shown to be incredibly useful and highly characterized through experimentation and thus should play a major role in preclinical research and its translation into clinical success.

### 9.2. Radial Arm Maze

**Figure 17.**
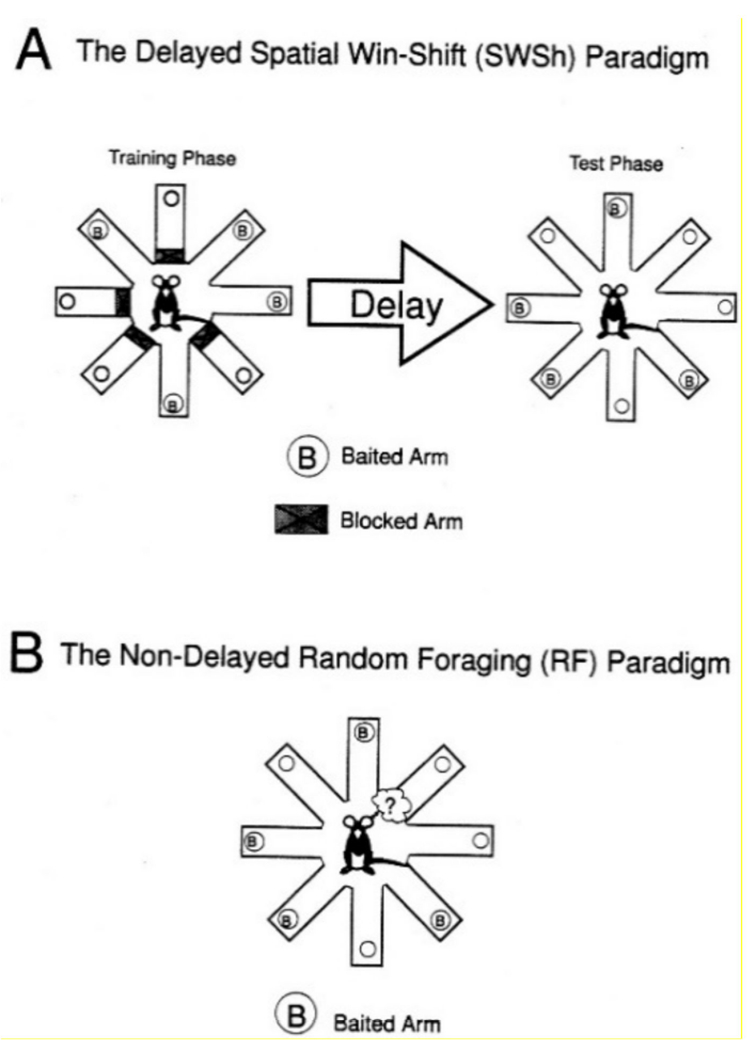
Example of the RAM in both the (**A**) delayed win-shift paradigm, where four of the eight arms are blocked then, after a delay, are opened with bait placed in the formerly blocked arms and (**B**) the non-delayed paradigm, where no delay is present a random set of four arms are baited. Reproduced with permission from [84].

The Radial arm maze (RAM) is an eight-armed, walled maze, although variations in the specific number of arms exist. Pre-trial starvation or dehydration is used so food and water can be used as a positive stimulus to encourage exploration (food or water placed throughout the maze) and learning (food or water placed at the end of each arm) [82,83]. Spatial learning and memory are tested using extra-maze visual cues to allow the animals to create a spatial pattern in their mind or to use nonspatial methods of determining how to most efficiently find all the food in the maze, such as turning only one direction. There are two major RAM paradigms: the delayed spatial win-shift and the non-delayed random foraging (Figure 17). These paradigms have multiple different characteristics, including the former using arm blocking and two phases, while the latter uses only one phase. Both paradigms bait half of the arms to test learning. While spatial cues are not necessary, they are required to shift this from simply a learning paradigm to specifically a spatial learning paradigm. For a more comprehensive look at a particular protocol, Floresco et al. have provided a comprehensive explanation [84].

Each paradigm produces different specific datasets. The delayed paradigm data are primarily taken from the second part of the test after the delay. At this time, errors are counted as entries into arms that had not been previously blocked during the training phase. Additionally, errors are split into two groups, across-phase and within-phase, which are more thoroughly described in Table 2 [84]. The non-delayed paradigm includes only the single trial of testing and describes errors much more broadly as any re-entry into an arm, whether that arm contains bait or not. However, these are also broken down into two subtypes: re-entries into arms that had been baited at the beginning and re-entry into arms that had not been baited [84]. Both paradigms share total latency and first latency despite their differences. While several types of data can be obtained using this, clinical translation is often very difficult.

Similarly to the MWM, clinical researchers have used VR RAM paradigms to attempt to connect preclinical work with clinical testing. Much like the MWM, the VR paradigm for the RAM shows similarities to results observed in rats. For example, clinical research has been able to demonstrate that the usage of spatial and nonspatial learning corresponded with activation of the brain regions controlling the two forms of learning, namely the hippocampus and caudate nucleus, respectively, which is also observed in rats [79].

### 9.3. T and Y Maze

**Figure 18.**
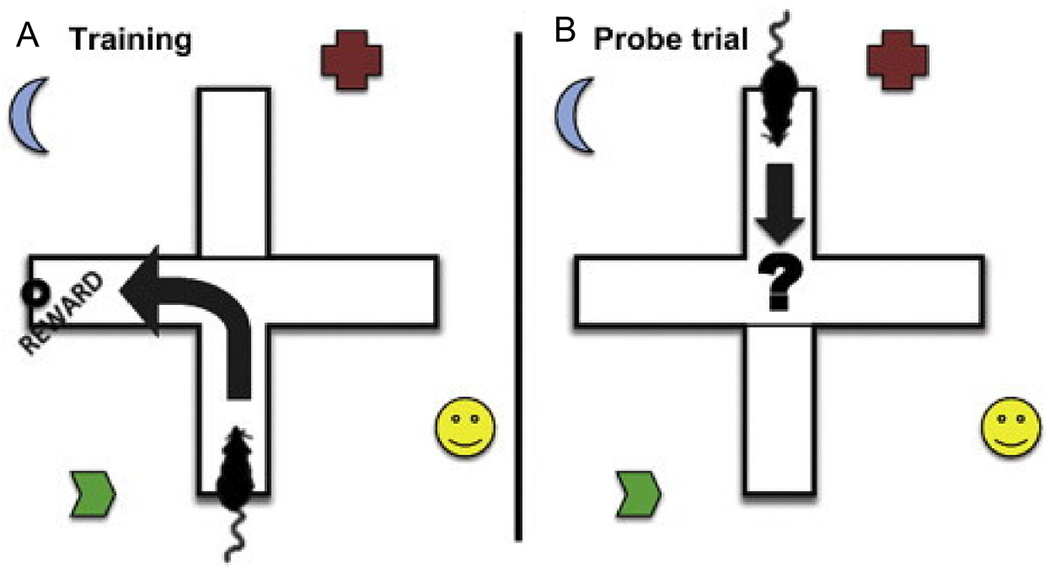
Example of a T maze paradigm, the dual-solution T maze, used to measure both place and response learning. (**A**) The training phase shows a reward placed one left turn from the rodent. (**B**) On the probe trial day, the reward is removed and direction turned (left versus right) shows nonspatial and spatial learning, respectively. Reprinted with permission from [79]. Copyright 2013 Elsevier.

T and Y mazes are similar, based on the same principle of spatial learning and memory. Both mazes function as a two-pronged maze using either positive stimuli (e.g., food, novel objects) [85–87] or negative stimuli (e.g., light, electrical shock and sound, a blocked arm) [88,89] to promote memorization of the different arms. After training, the stimuli are removed, and animals are tested again to measure memory. Additionally, some variations of the T maze use distal spatial cues to help promote learning and to determine spatial learning in a similar fashion as the MWM and BM tasks [88]. One variation utilizes both positive stimuli during training and spatial cues in a combined system. In this variation, mice are tested for two forms of spatial learning, place learning and response learning (Figure 18) [79]. Place learning can be described as the utilization of spatial cues to determine location, while response learning can be described as using internal cues such as the direction of a particular movement. For example, the animal would be using place learning if it turns toward the reward during the probe trial and response learning if it turns away from the reward. Essentially, place learning and response learning can be equated to spatial learning and nonspatial learning, respectively.

The T and Y maze offer very few data, even with the dual-solution T maze described (Figure 18), which can distinguish between place and response learning in the rodent model [90]. The alternating T maze, which utilizes two phases involving a training phase where one arm is blocked, measures time spent in the unblocked, or novel, arm as a percentage of total time spent in the maze. While this measurement is a general measurement used in most T and Y maze testing despite the version, the alternating T maze also uses forced alternation as a data point [91], which is described in further detail in Table 2.

The T and Y maze have a less significant clinical connection when compared to the VR MWM or RAM. These issues stem from the simplicity of the maze, which is ironically one of the reasons these can be such popular mazes. These mazes have the same issues that plague others, specifically the lack of motivation in humans [79]. Humans do not have the same motivations in VR as animal models do in preclinical testing, such as the potential for drowning, starvation, or even minor annoyances such as the strong lighting in the BM. Therefore, human patients require some outside source to provide a stimulus while the test is taken in VR, such as food or monetary rewards. Regardless of other methods to increase virtual T maze viability, the MWM and RAM VR tasks seem to show much more promise as a viable connection between the preclinical and clinical sides of testing.

### 9.4. Novel Object Location Test

**Figure 19.**
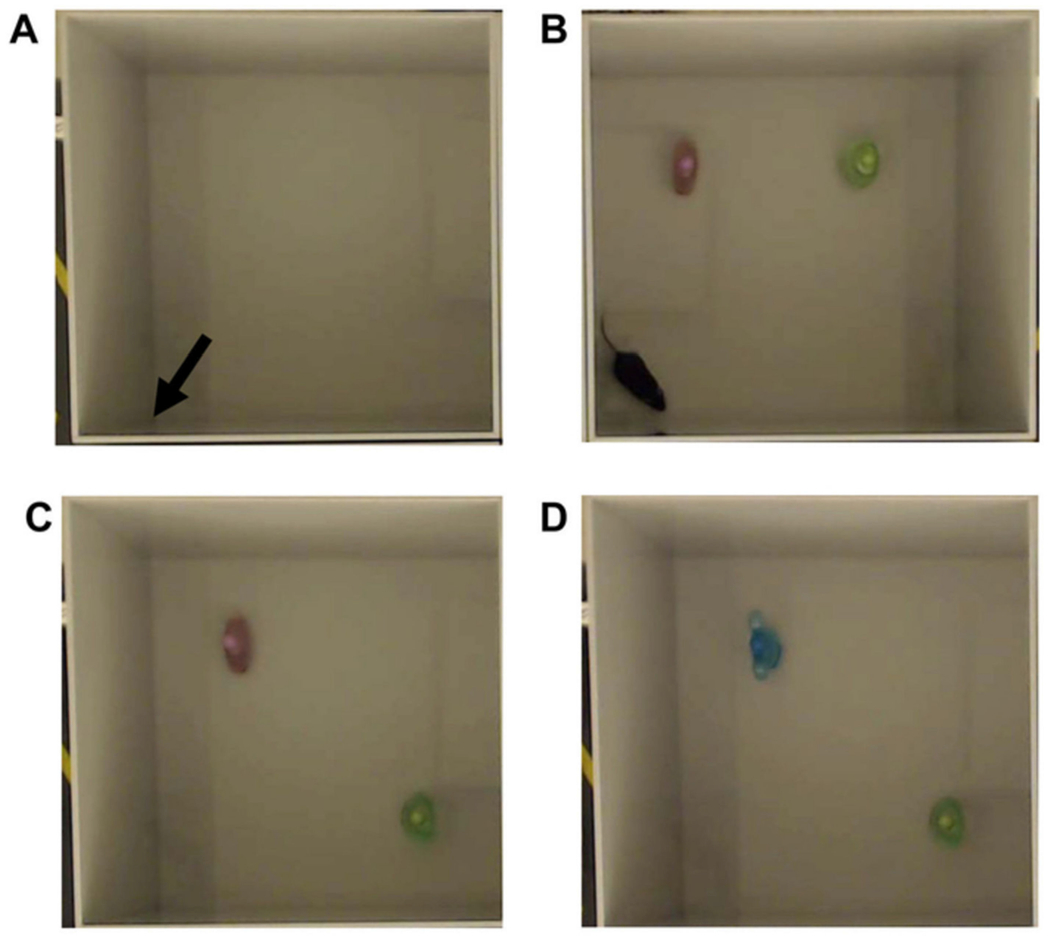
Examples of the Novel Object Location (**A**) for habituation, (**B**) for training, (**C**) for location change) and Novel Object Recognition (**A**) for habituation, (**C**) for training, (**D**) for object replacement). Panel (**A**) also represents an example of the open field test, described in the section on emotional tasks [94]. Adapted with permission from [94]. Copyright 2018 MyJoVE Corporation.

In the Novel Object Location test (Figure 19), rodents are allowed to explore an empty open field for 5 min. Animals are then given a 5 min trial one hour later with the objects placed in the open field and then another 5 min trial one hour later with one object in the same place and another object in a new place within the field [79,92]. The one-hour inter-trial interval forces the animal to rely on the long-term memory rather than short-term memory or luck. Rodents are expected to use their natural curiosity to spend more time examining the object in a novel location as opposed to the object which had not moved. However, deficits are shown when animals chose to explore both objects similarly to the middle phase prior to object relocation, showing an inability to remember the familiar location when faced with a novel location.

The Novel Object Recognition task is a nonspatial variation of the Novel Object Location task. In this test, rather than one of the same two objects being moved to a new location, the object is instead replaced with a new object the animal is unfamiliar with. Similarly to the Novel Object Location task, it is expected that TBI animals will spend a near equal time exploring both objects while uninjured animals will spend more time exploring the novel object [92].

At this time, human equivalents are only connected to the delayed non-match to sample task, which itself is a behavior test used with animals already [93]. This separate test is administered by giving the subject an initial set of stimuli, generally a set of objects, and providing a separate, novel object after a delay and requiring the subject to select the novel stimulus [93]. The changing of objects can create a thorough connection to the Novel Object Recognition task; however, this is considered to be more similar to the delayed match to sample task as there seems to be some correlation between the slightly different mechanisms of memory used in each task.

Both tasks share data similarities, as time spent with the novel object or location in terms of a fraction of time spent in the maze are the primary data point of measurement. However, a metric called the discrimination index is also used and measured by subtracting the time spent exploring the familiar location or object from the time spent with the novel location or object divided by the total time exploring either object. It is important to note that this does not mean the total time spent in the open field but rather the summation of time spent exploring either object or location [94].

## 10. Nonspatial Learning and Memory

As opposed to allocentric navigation, as described above, egocentric navigation is a method of determining how to travel similarly to how one might go about a traditional maze, using memory of motions made in conjunction with interior focal points to map out the area mentally. This kind of navigation can be seen in patterns such as the serial and non-spatial navigation shown in the BM and MWM (Figures 14 and 15). While this can occur in many spatial learning tasks such as the RAM, certain variations of spatial learning tasks can be altered to examine nonspatial learning and memory specifically. While the overall administration of these tasks changes for the preclinical models, clinical delayed non-match to sample and VR tasks can also be adjusted to similar specifications to test nonspatial learning and memory.

### Spatial Learning Task Variations for Nonspatial Learning

Many paradigms such as the RAM, MWM, and BM can test for nonspatial learning. Indeed, in each task, there are methods with which nonspatial learning can be examined without changing the protocol. Nonspatial search strategies can be present in each task, such as serial exploration in the RAM and BM and MWM strategies that show knowledge of the existence of an escape without a direct understanding of how to get there. Such strategies include serial strategies for the BM, random, focal incorrect, and scanning strategies for the MWM, and chaining or serial strategies in the RAM [74,75,95]. However, for researchers interested in limiting these to only nonspatial navigation, several methods have been explored, with the most common being to “drown out” or remove any extra-maze cues. Nonspatial navigation targets a different area of the brain when compared to spatial navigation. Particularly, the area which is most considered to dominate spatial navigation is the hippocampus, while the area most correlated with nonspatial navigation, also thought to be heavily implicated in the same areas as spatial navigation, implicates other brain regions such as the caudate nucleus and entorhinal cortex [96]. While nonspatial learning is a large field within neuroscience, its reasoning is less understood when compared to spatial learning, and therefore, it is less effective when determining differences between injured and uninjured animals or patients.

## 11. Emotional Tests

Emotional changes in human TBI have been well documented. Despite this, many of the emotional tests used to determine emotional deficits, such as anxiety-like behaviors, lead to directly conflicting results depending entirely upon the paradigm, even within the same procedures. These differences have yielded results determining both high and low levels of anxiety in the same open field test along with equal anxiety when compared to uninjured counterparts [97]. Many of these tests yield similar conflicts in TBI research. Additionally, human patients have reported near day-to-day variability in their levels of anxiety, depression, and other emotional markers [98]. This may influence attempts to find correlations between preclinical studies of TBI and clinical studies. However, many of these models have been used for drug exploration in other realms such as antidepressants, antianxiety, and other various psychopharmacological drugs. This may redeem some of the criticisms these tasks have been given in the realm of TBI research, though the innate variability of emotional deficits in TBI could also account for that difference.

### 11.1. Forced Swim Test

**Figure 20.**
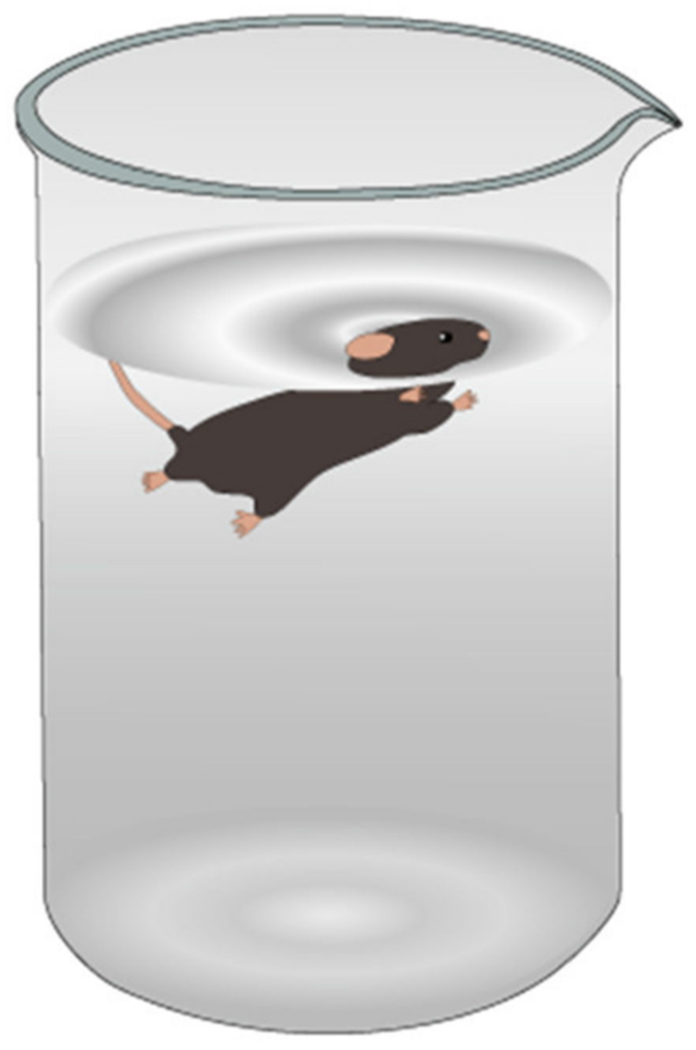
Example of the forced swim test. Image by DataBase Center for Life Sciences, distributed under a CC-BY 2.0 license.

The forced swim test was designed originally for testing of antidepressant drugs and is accepted as a preclinical model of depression because of its usage in testing for antidepressant medication [99]. The protocol for this test requires a 10 cm diameter transparent cylindrical tank filled with water to 15 cm from the bottom (Figure 20). Both diameter and depth can be altered to change behavior, such as the length of time mice were willing to maintain struggle by continuing motor activity which increased with larger tank diameter and deeper water [100]. These conclusions, while important in the field of anti-depressant testing, have less importance within the field of TBI testing, where, for the sake of the effects of TBI on depression, the standard depth and tank width provide sufficient information to researchers. It is worthy to note that the testing performed by Sunal et al. found that larger tanks with a longer duration, namely 15 min, may provide a more accurate measurement without as many issues of false positives [100]. The water should be room temperature and rodents should be placed in the tank gently and remain there for six minutes. Intervention in the test should only be carried out if the rodents cannot maintain swimming or floating, or, in a special case with mice, any diving behavior is observed [99].

The data derived from these experiments have three basic components: time spent inert, time spent climbing, and time spent struggling. While an animal is climbing, it is attempting to come up the side of the vessel of water. While an animal is struggling, it is making active movements to try and stay afloat or get out of the water. While an animal is inert, it is making no movement and can thus be considered as an act of despair, similar to depressive-like symptoms in humans. The major data point for this test is the time spent inert, which can be interpreted as depressive-like symptoms.

### 11.2. Dark/Light Avoidance Test

The light/dark avoidance test is used to quantify anxiety-like behaviors. Rodents have a natural aversion to well-lit areas, as referenced when discussing the BM. The light/dark test utilizes this as a way to determine anxiety-like behaviors by defining the light area as an anxiolytic zone and measuring time spent in the light and dark zones along with path length in each zone over a 15 min period [101].

The major data gathered from this experiment are the time spent in both dark and light zones, the distance travelled in both zones, the time it takes to visit the light zone for the first time, as well as the number of entries into the light zone in total [101]. Each of these measurements show a higher level of anxiety if more time and distance are spent in the dark zone as well as if the latency to the light zone is higher and number of entries is lower.

### 11.3. Open Field Test

The open field test is useful for measuring both locomotion and anxiety-like behaviors in rodents and is one of the most commonly used methods of behavioral testing, especially in rodents. The field (Figure 19A) consists of a walled area with a light focused directly above the area with a 10 min limit to the test. For anxiety testing, measurements of time spent in the outside area of the maze, known as thigmotaxis, are considered to be a marker of anxiety-like behavior. The more time an animal spends in the center of the arena, the less anxiety-like the animal’s behavior. Additionally, movement can be measured with higher amounts of distances travelled being considered as an anxiety-like reaction [102].

### 11.4. Resident Intruder Test

The resident intruder test is a common test for aggression. Much of the data gathered from this test are specifically behavioral, relying heavily upon noticing differences, frequency and duration of offensive aggression, defensive aggression, and violence. Each of these categories have well-defined parameters as described by Koolhaas et al. To establish territoriality with rodent models, a male is housed with a sterilized but hormonally intact female companion for at least one week. During the test, the female is replaced with a novel male into the cage and observed to determine a battery of scoring measuring two opposites of behavior, aggression and sociability/anxiety, measured by the Total Offense Score and the Social Exploration Score, respectively [103]. Additionally, latency to first attack is also an often-used measurement to determine aggression with lower latency corresponding to a higher amount of aggression. This protocol can also be adjusted for female mice with almost no change, except to make sure female companions are age-matched to avoid conflict [104].

## 12. Motor Coordination

Motor coordination tasks, otherwise known as vestibulomotor tasks, measure the coordination and physical differences between injured and uninjured rodents. These are the most easily transitional tasks between clinical and preclinical studies as human TBI has been shown to cause adverse effects, at least acutely, to motor coordination and cognition [105].

### 12.1. Rotarod

**Figure 21.**
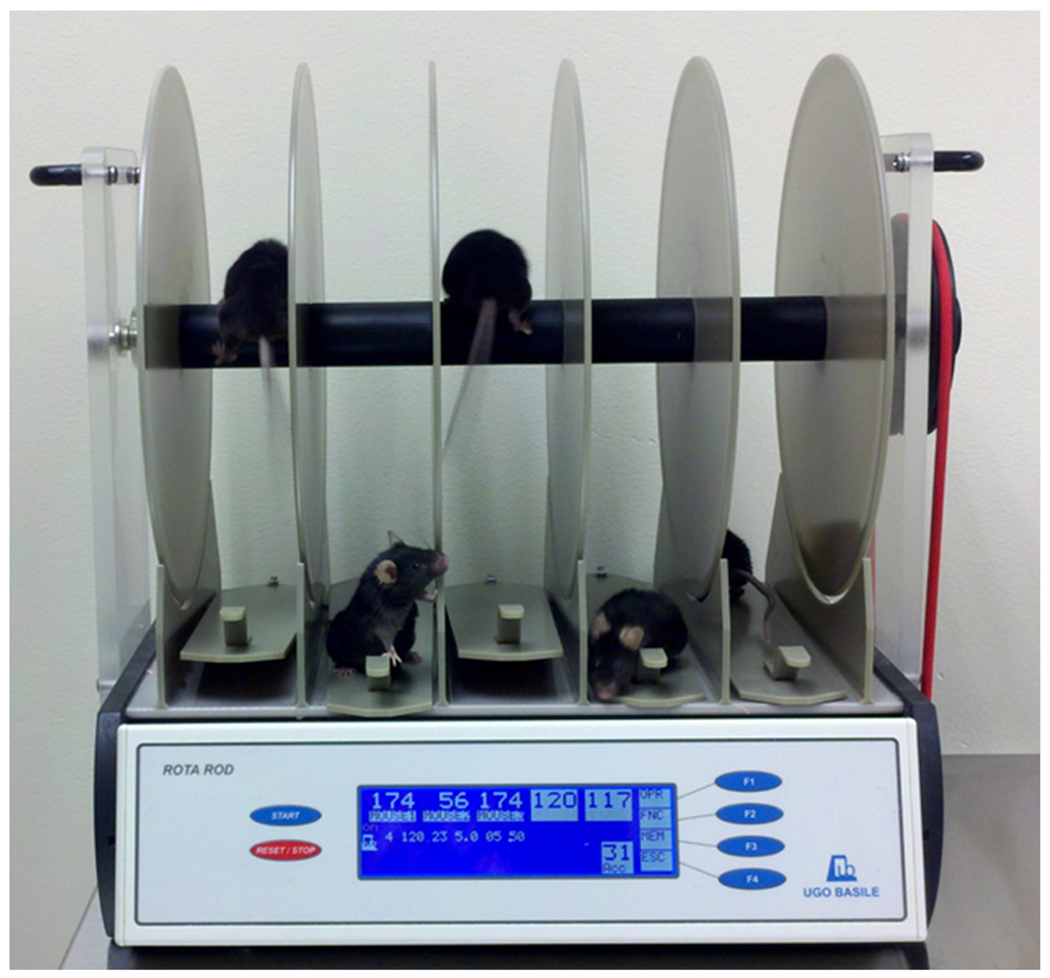
Example of a Rotarod machine used to measure motor coordination deficits. Image from Bmouzon, distributed under a CC-BY 2.0 license.

The rotarod test is a widely used test to determine coordination deficits in rodents. A linearly accelerating cylinder that animals are placed on continues to rotate until all animals have fallen or until the final time point is reached (Figure 21). This is most effective for motor deficits in the acute phase of injury, but may also be used later prior to cognitive testing to ensure there are no motor deficits when using methods such as the MWM, RAM, or other spatial or nonspatial learning tasks. Latency to fall is the most important measurement with this method; however, qualitative analyses can include coordination by way of the method with which the animal stays on the rotarod [106,107].

### 12.2. Open Field Test

The open field test, as described above, is commonly used for both anxiety testing and motor coordination. When used for motor coordination, the above-described methods are still used, but different measurements are taken. Data for this test include distance moved, time spent walking and running, slower or hyperactive movements, jumping, rearing, and other rodent behaviors described previously. However, the most used and understood data point for motor coordination is the distance travelled [102]. Depending on the timing of this test, one should expect slower movement in TBI mice in the acute phase and more hyperactive movements in the chronic phase, as well as a lower distance moved and higher distance moved for TBI mice in the acute and chronic phases, respectively [107]. Additional information regarding the open field test as both a method of measuring motor coordination and anxiety-like behavior are described in Table 2. Along with the rotarod test, this test is highly characterized and accepted by the behavioral testing community.

### 12.3. Footprint Pattern Assay

The footprint pattern assay is executed by dipping a rodent’s paws in different ink colors for the fore and hind paws and leading them down a tunnel lined with paper. Through this method, abnormalities in gait and coordination can be observed. Additionally, many parameters are capable of being measured, such as stride distance, stride length, variability across the center axis of the paper, width between hind paws, step regularity, and step overlap. Many of the most important aspects of the footprint assay include the step length, step duration, and inter-leg coordination, as described in Table 2 [108]. Modernized versions of this assay are automated and also capable of measuring pressure and speed, such as the CatWalk™ system [109–111].

## 13. Comparison of TBI Animal Models to Human Injury

Due to the heterogeneity of physiological outcomes experienced following TBI in humans, defining a clear diagnosis for a given individual can be difficult and often ambiguous. Categorizing TBI using classification systems alleviates some of the uncertainty by determining the level of injury based on a variety of factors analyzed following the initial impact. However, conducting a proper diagnosis for an individual’s TBI severity is only a portion of the challenge when combating the condition. Medical advancements for the treatment of TBI require experimentation using animal models to ensure methods are effective and safe for treating patients. Therefore, classification systems and animal models must be used in conjunction to differentiate between different levels of injury severity and improve medical care.

Based on the classification methods previously discussed, injury severity in humans can be defined based on the following factors: injury mechanism, presence of major extracranial injury (MEI), GCS, and imaging characteristics. Unfortunately, classifying animal models using the GCS would be difficult due to the limitations in the examination criteria. However, there are additional cognitive and behavioral tests that could be used to classify these animal models, including the MWM and BM discussed previously. Establishing a definitive behavioral assessment for characterizing animal models would be a useful tool for increasing comparisons between animal models and clinical TBI. Table 3 shows the categorization of each of the animal models described previously based on their method of impact and physiological outcomes. Models were classified based on the categories from the CENTER-TBI results; however, mild and upper intermediate TBI levels were grouped together due to strong similarities when comparing the mechanism of injury and imaging characteristics. Many of the animal models in these categories would be ideal for producing mild or upper intermediate injuries by adjusting impact factors such as the height of the weight dropped in the Marmarou weight drop model and the pressure pulse in the midline FPI [14].

## 14. Perspective and Recommendations for the Nanotheranostics Researcher

Extensive research has been established into the mechanisms of damage following primary brain injury; however, there are still key elements that need to be discovered for the development and progression of treatment options following TBI. Difficulties surrounding TBI intervention include injury heterogeneity of the patient population and optimization of treatment accumulation in damaged regions of the brain. Recent literature has focused on the development of nanoparticles for use as theranostic tools for the diagnosis and treatment of TBI. Previously developed animal models and behavioral tasks have aided researchers in identifying the efficacy of treatment options for alleviating biochemical malfunctions and cognitive deficits following TBI. However, prior to investigating the efficacy of nanotheranostic intervention, several considerations must be addressed regarding animal models and behavioral tasks.

Several animal models have been established for identifying changes in brain structure, biochemical markers, and cognitive behavior following primary injury. Additionally, while the animal models mentioned in this review are primarily referencing the usage of rodents, many of these models have incorporated various species in their research, including ferrets, cats, rabbits, dogs, sheep, swine, zebrafish, and flies [14,112,113]. Each of these models provides unique strengths and weaknesses for producing the desired pathophysiological consequences and cognitive deficits. Focal injury models will be more suited for producing moderate to severe injuries with notable structural damage and hemorrhaging, while diffuse injury models will be more effective at mitigating lesions and creating diffuse axonal injury. Additionally, considerations regarding the injury severity, mechanism of injury, and reproducibility must be addressed. Prior to incorporating an animal model for assessing the efficacy of nanotheranostic tools, researchers must first consider the level of injury severity desired for their experiment. Based on this review, injury severity falls into mild, upper intermediate, lower intermediate, and severe categories, which correspond to the level of damage produced following impact. For early research, establishing evidence for the accumulation and therapeutic intervention of nanoparticles would be most efficient in a severe injury category of TBI. Severe brain trauma produces the greatest alterations in the neurological structures of brain tissues, biochemical markers, and cognitive behaviors, allowing for the greatest difference between uninjured control animals. These injuries would show the greatest comparison for passive accumulation of nanoparticles, due to the dramatic alterations in brain structure. In contrast, milder models of TBI would provide a more rigorous environment for assessing and confirming active accumulation in the region of interest. Additionally, care must be taken to not utilize too severe of an injury where treatment would not make a large impact on outcome. Indeed, care should be taken to avoid a full destruction of the brain regions being tested, such as the hippocampus for spatial learning. Using the hippocampus as an example, research has found that the dorsal hippocampus is important for spatial learning and memory bilaterally; however, the right hippocampus seems to be more useful in split-brain models, implying that the right hippocampus has a larger effect on the accuracy of spatial memory [73]. With this knowledge, it is important to consider the side of injury, the severity of injury, and the measurements being utilized during behavioral research. It is also important to maintain some amount of brain structure on the impact side. For example, moderate-to-severe CCI to the left cortex with a 2.5 mm depth will cause some mechanical damage, though not complete damage, of the left hippocampus. Additionally, this model can measure secondary injury severity indirectly through behavioral changes. Untreated mice should have decreased accuracy for spatial learning and memory due to the spread of secondary injury, with treatment causing an increase in accuracy and therefore a decrease in deficits. Injury severity is an incredibly important parameter that should not be considered lightly. Behavioral effects and even secondary injury severity can all be determined based on the severity of the primary injury, as well as which model was used to cause the primary injury.

Once the level of injury severity desired has been identified, the focus shifts towards the mechanism of injury. Mechanism of injury refers to the type of impact causing the production of damage leading to TBI, and how this mechanism of impact translates from animal model to features of clinical TBI. The mechanisms of injury discussed above in this review include focal, diffuse, and non-impact injuries, corresponding to a variety of different pathophysiological features. Elements such as cortical tissue loss and DAI would be more pronounced in focal and diffuse injuries, respectively. Lastly, considerations must be made regarding the reproducibility of the animal model. Elements of reproducibility include surgical intervention prior to impact, capabilities for adjusting injury severity in the desired animal model, in addition to mortality rates following injury. Surgical intervention includes craniectomies and the succession of artifact placement inside the cranial window. Several models require scalpel incision followed by craniectomy prior to injury, which can negatively impact reproducibility if there is damage to the underlying dura mater. Additionally, craniectomies and artifact placement may require additional equipment and training, which may not be desirable in early research and development. As mentioned previously, craniectomies should not exceed the cranial defect size for the given animal, which limits the size of the overall impactor in certain models. Lastly, it is important to incorporate a sham model when conducting research that requires a craniectomy or artifact placement. This will be vital in separating the effects from injury and surgical perturbation of the skull. In addition to surgical intervention, model adaptability regarding injury heterogeneity may also be a useful consideration. Animal models that can be adjusted to produce varying levels of injury severity are useful when considering the efficacy of nanoparticles across a wider range of clinical TBI presentations. For example, several adaptations have been developed for the weight drop method to create injuries in mild, moderate, and severe categories, while it would be difficult for the PBBI method to reproduce a mild injury. Additionally, the CCI model utilizes precise mechanical adjustments for manipulating elements of injury, similar to the CHIMERA model, which may be useful for creating a variety of injury intensities. This element of adaptability would be useful for the continuation of a single animal model for long-term TBI research. Additionally, mortality rate is an important element to consider following injury. A few of the animal models discussed here have been shown to reproduce high percentages of animal mortality depending on the severity of injury and may require intervention following impact. Each of these considerations and recommendations must be analyzed prior to selecting an animal model useful for assessing nanotheranostic tools for the diagnosis and treatment of TBI.

Identifying and comparing cognitive alterations following primary injury produced from these variations of animal models requires the use of behavioral tasks. Each behavioral task is designed to assess specific cognitive functions and will be affected by damage to different regions of the brain following injury. Behavioral tasks must be chosen with careful consideration of one major factor: injury phase. It is our recommendation that a prospective nanotheranostic researcher begin by first determining the validity of their TBI model in the acute phase with a paradigm well established for motor locomotion, such as the Rotarod task. This is important as it shows that the chosen parameters and injury model are causing deficits. However, it is not necessary to begin therapeutic testing, as the effects of therapeutics on secondary injury at this point are minimal. However, as the response to the nanotheranostic agent is well categorized for the acute phase, it is important to consider different paradigms more useful in the subacute and chronic phases to determine therapeutic efficacy. For both subacute and chronic phase testing, it is recommended that one uses a highly characterized spatial task such as the MWM. Indeed, the MWM is a leading standard in neuroscience research to determine spatial learning and memory and can be easily employed for chronic phase testing. It is important to note that the MWM may also be used in the subacute phase; however, locomotive deficits must be confirmed to not be occurring for results to be considered valid.

## 15. Conclusions

TBI is currently the leading cause of morbidity and mortality for children and adults under the age of 45 due to the variety of circumstances capable of producing head trauma. While medical advancements have improved the methods for diagnosing and treating patients with TBI, the progression of secondary injury led by mitochondrial dysfunction, glutamate toxicity, oxidative stress, and a variety of additional biochemical complications have continued to create issues for medical personnel. Additionally, TBI presents with a multitude of physical, cognitive, and behavioral deficits which vary between individuals depending on a combination of multiple factors, including injury severity and mechanism of injury. Due to this variability in the TBI population, classification methods have been developed for categorizing patients into specific levels of injury. While GCS has become an effective and efficient tool for classifying TBI severity, new classification systems such as Mayo Clinic’s model and the results from CENTER-TBI have shown the benefits of incorporating a variety of characteristics. These primary factors include injury mechanism, presence of major extracranial injury, GCS scores, and imaging characteristics. Current research has begun developing a prediction model for the progression of TBI, which would play a key role in the diagnostic process. In addition to classification methods, animal models have been developed for experimentation to ensure treatment options are effective and safe. These models are divided into three specific subsections, namely focal, diffuse, and non-impact injury, which are beneficial for characterizing the type of impact in the model. However, some models incorporate multiple elements, which increases reproducibility and reduces key limitations such as mortality rates. These designs, including the modified Marmarou weight drop model and the modified CCI model, provide a broad range of advantages for the user which could be beneficial when collecting data and conducting analysis. Categorizing the animal models based on previously established classification systems would provide additional framework for researchers to compare between the different models. Additionally, classifying the animal models creates an additional comparison to human TBI, ultimately benefiting diagnostic and treatment methods. In the future, effort should be placed towards establishing a standardized behavioral assessment for comparing animal models, in the hopes of effective translation between cognitive deficits seen in animals and humans. Including behavioral analysis would further strengthen the comparison between animal models and human TBI, leading to increased success in clinical trials.

## Figures and Tables

**Table 1.** Assessment criteria of the Glasgow Coma Scale used for determining injury severity in a clinical setting [21–23].

Response	1	2	3	4	5	6
**Eye**	None	To Pain	To Speech	Spontaneous	N/A	N/A
**Verbal**	None	Incomprehensible Sounds	Inappropriate Words	Confused Conversation	Oriented	N/A
**Motor**	None	Extension (Decerebrate)	Abnormal Flexion (Decorticate)	Withdrawal (Normal Flexion)	Localizes Pain	Obeys Commands

**Table 2.** Key data generated from the behavioral paradigms discussed in this review, their interpretation, and their expected changes following TBI.

Behavioral Task	Data Type	Description	Expected Result (Compared to Control Group)	Meaning of Results
Spatial Learning and Memory				
MWM	Latency to Platform (s)	The amount of time it takes an animal to escape the maze.	TBI should take longer	Decreased latency shows a higher amount of spatial learning.
	Percent in Quadrant (% or fraction)	The percentage of time spent in a specific quadrant over the total time in maze.	TBI should spend less time near the escape and more time in quadrants away from the escape	High percentages in the quadrant of the platform show higher learning; however, high percentages in the reversal week in the former escape quadrant show an inability to relearn.
	Percent of Time in the Outer Annulus (% or fraction)	The percentage of time spent in the outer annulus of the maze.	TBI should spend more time in the outer annulus	Higher percentages in the outer annulus show thigmotaxis, which shows no learning or confusion.
	Path Length (cm)	The length of the path made while moving through the maze.	TBI should have a large path length	Higher path length shows more movement and a lower understanding of how to escape the maze and thus, less ability to learn and memorize the maze.
	Cumulative Distance from the Platform (cm or m)	The distance, measured every few seconds or milliseconds, from the platform.	TBI should have a larger cumulative distance	Longer distances show a lack of spatial or non-spatial search strategies, which indicate worse learning or memory.
	First Bearing (Degrees or radians)	The angle between the first movement of the animal and a direct line to the platform.	TBI should have a larger degree of first bearing	Higher degree of first bearing shows a deficit in memory of where the platform lies spatially.
	Search Strategy	The strategy (i.e., spatial, nonspatial, or random) the animal uses to find the platform.	TBI should use more random or nonspatial strategies	Higher use of random search strategies indicates lower learning and memory while the inverse of higher spatial strategies shows an increase in learning and memory.
	Probe Trial Time in Target Quadrant (% or fraction)	The time spent in the quadrant where the platform should be as a percentage of total time.	TBI should spend less time in the target quadrant	Higher percentage of time in the target quadrant shows an increased ability in learning and memory of the maze.
	Probe Trial Platform Crossings (Frequency)	The number of times the area where the platform should be is passed over.	TBI should pass over less	Higher frequency of platform crossing shows better learning and memory.
	Swim Speed (m/s)	The velocity at which animals are travelling in the maze	TBI should be relatively similar in order to rule out motor deficits; however, this is specific to post-acute phase testing	Lower swim speed shows either a motor coordination deficit, or, potentially but unlikely, a lower ability to learn and remember the maze. These should, in most circumstances, be very similar.
BM	Primary Latency (s)	The amount of time it takes an animal to find the escape and enter (head only).	TBI should take longer	Lower primary latencies show a better understanding of the escape and how to reach it via nonspatial navigation or spatial navigation, depending on search strategy.
	Total Latency (s)	The amount of time it takes an animal to find and fully enter the escape hole.	TBI should take longer	Lower total latency shows learning and memory into which method will provide escape the quickest.
	Reference Errors (Frequency)	The number of times an animal enters a non-escape hole with its head.	TBI should have more errors	Higher reference errors show a decreased ability to learn and memorize the maze.
	Working Errors (Frequency)	The number of times an animal makes a reference error after having visited that hole before.	TBI should have more errors	Higher working errors show a decreased understanding of the maze along with potential confusion regarding visited areas, showing a lack of memory.
	Perseverative Errors (Frequency)	The number of times an animal repeats searching the same hole before moving on to another.	TBI should have more errors	Higher perseverative errors show a lack of learning and memory of places previously visited and may, in reversal trials, indicate an inability to relearn.
	Primary Errors (Frequency)	The number of times an animal enters a non-escape hole with its head before finding the escape hole.	TBI should have more errors	Higher primary errors indicate deficits in learning and memory of the maze.
	Total Errors (Frequency)	The number of times an animal enters a non-escape hole with its head before entering the escape hole with its whole body.	TBI should have more errors	Higher total errors indicate deficits in learning and memory of escape of the maze, or, when combined with low primary latency, more curiosity from the animals, indicating comfort in the maze.
	Hole Deviation Score	The number of non-escape hole visits between the first visited hole and the escape.	TBI should have a higher score	Higher hole deviation scores show a lack of learning and memory when related to finding the correct path in the maze. Spatial learning will show lower scores than nonspatial learning.
	Primary Path Length (cm)	The distance an animal has travelled before reaching the escape hole with only its head.	TBI should have a longer distance	Path length, in either context, shows a decreased ability to understand and memorize the maze.
	Total Path Length (cm)	The distance an animal has travelled before entering the escape hole with its whole body.	TBI should have a longer distance	Path length, in either context, shows a decreased ability to understand and memorize the maze.
	Search Strategy	The strategy (i.e., direct/spatial, serial, or mixed/random) the animal uses to find the escape hole.	TBI should use more mixed/random strategies and fewer direct/spatial strategies	Higher use of mixed/random search strategies show a decreased ability to learn the maze; however, an increase in serial strategies after a large number of spatial strategies show complacency within the maze
	Velocity (cm/s)	The change in distance over time at which animals are travelling in the maze.	TBI should be similar during chronic phase, acute phase measurements may be lower for TBI	Lower velocity can indicate motor coordination issues within the maze. These should stay relatively similar throughout both weeks of trials.
RAM	Errors (Frequency)	For delayed test, the number of entries into non-baited arms. For the non-delayed, re-entries into the arms entered previously that trial.	TBI should have more errors	Higher frequency of errors shows a lack of memory,
	Across-Phase Error (Frequency)	Entry to an arm previously entered during the training phase (delayed test only).	TBI should have more errors	Higher frequency of these errors shows a poor ability to learn from the training phase and thus a worse long-term memory,
	Within-Phase Error (Frequency)	Entry into an arm entered within the test phase (delayed test only).	TBI should have more errors	Higher frequency of these errors shows a poor ability to remember what has been visiting, showing a worse short-term memory,
	Baited Arm Re-entry (Frequency)	A second entry into an arm that had been baited at the beginning of the trial but was already discovered (non-delayed test only).	TBI should have more errors	Higher re-entries of this type show a lack of learning.
	Non-baited Arm Re-entry (Frequency)	A second entry into an arm that was not baited at the beginning of the trial but was already discovered (non-delayed test only).	TBI should have more errors	Higher re-entries of this type show a lack of memory.
	First Latency (s)	The time it takes for the animal to first visit a baited or non-baited food cup.	TBI should take longer	Higher first latency shows a hesitancy to explore the maze and potential deficits in memory or learning. This may also indicate a nonperformer.
	Total Latency (s)	The time it takes for the animal to retrieve all food pellets.	TBI should take longer	Higher total latency shows a lack of learning and memory.
T and Y Maze	Time Spent in Novel Arm (% or fraction)	The amount of time the animal spends in the opened arm during the second trial (alternating T/Y maze only).	TBI should spend about equal time exploring both arms	A lower percentage of time spent in the novel arm shows memory deficits.
	Forced Alternation (% or fraction)	The percentage or fraction of animals that enter the novel arm first during the second trial (alternating T/Y maze only).	TBI should enter the novel arm less	A lower percentage of forced alternation shows a lack of learning.
	Place Versus Response Learning	When the direction of the entrance arm is switched, the animal will either use spatial learning and turn toward goal or nonspatial learning and turn the direction turned during training.	TBI should more often use nonspatial learning and turn in the direction it did during training	This shows the difference between place learning (spatial learning) and response learning (nonspatial learning).
Novel Object Location	Percent of Total Investigation Time (% or fraction)	The time spent exploring the novel location divided by the total time spent exploring either object.	TBI should spend about 50% of the time or less exploring the novel location	A lower percentage of novel investigation shows an inability to remember the familiar object.
	Discrimination Index	The time spent exploring the novel location minus time spent exploring the familiar location divided by total time exploring either object.	TBI should be closer to zero; positive values show more time investigating the novel location	A higher discrimination index shows a preference to explore the novel object rather than the familiar object.
Nonspatial Learning and Memory				
Novel Object Recognition	Percent of Total Investigation Time (% or fraction)	The time spent exploring the novel object divided by the total time spent in the exploring either object.	TBI should spend about 50% of the time or less exploring the novel object	A lower percentage of novel investigation shows an inability to remember the familiar object.
	Discrimination Index	The time spent exploring the novel object minus time spent exploring the familiar object divided by total time exploring either object.	TBI should be closer to zero; positive values show more time investigating the novel object	A higher discrimination index shows a preference to explore the novel object rather than the familiar object.
Nonspatial Variants of Spatial	Same data as described above	Nonspatial variants simply take away spatial cues for each task.	Refer to above corresponding expectation for spatial tasks.
Emotional				
Forced Swim Test	Time Spent Immobile (s)	The time spent not attempting to climb, move, or leave the swimming column.	TBI should spend a longer time immobile; however, depression-like activity is still controversial	A longer time spent immobile shows a larger number of depressive-like symptoms.
Dark/Light Avoidance Test	Time Spent in Either Zone	The time spent in either the light or dark zones. These will amount to complimentary measurements.	TBI should spend more time in the dark zone	Longer time spent in the dark zone shows a higher level of anxiety-like behaviors, while a longer time in the light zone shows the inverse.
	Distance Travelled in Each Zone (cm)	The distance travelled while in either the dark or light zone. This will also contain two separate data points for light and dark zones.	TBI should travel a greater distance in the dark zone	Higher distance travelled in the dark zone shows a higher level of anxiety-like behaviors, while a higher distance travelled in the light zone shows the inverse.
	Latency to Light Zone (s)	The amount of time it takes an animal to first explore the light zone.	TBI should take longer to explore the light zone	A greater latency to the light zone shows an increased amount of anxiety-like behavior.
	Number of Entries into the Light Zone (Frequency)	The number of times an animal enters and renters the light zone.	TBI should have fewer entries into the light zone	A lower number of entries into the light zone shows an increased amount of anxiety-like behavior.
Open Field Test	Time Spent in the Outer Zone (s or %/fraction)	The amount of time the animal stays on the outside of the open field, measured either as seconds or as a percentage or fraction of total time spent in the open field.	TBI should spend more time in the outer zone	A longer time spent in the outer zone infers an increased anxiety-like response to the open field.
	Time Spent in the Central Zone (s or %/fraction)	The amount of time the animal spends in the center of the open field, measured either as seconds or as a percentage or fraction of total time spent in the open field.	TBI should spend less time in the center zone	A higher amount of time spent in the central zone shows a decrease in anxiety-like responses.
	Total Distance Travelled (cm)	The distance the animal travels through the entire trial regardless of zone.	Differences could be from locomotor issues or a greater stress response from a change in general activity. It is important researchers take notice when using this measurement.	Total distance travelled should, normally, be relatively similar. However, a greater total distance travelled along with a significantly larger time spent in the outer zone may show increased anxiety-like behaviors. Additionally, decreased total distance travelled along with a significantly greater percentage of time spent in the center may show a decrease in anxiety-like behaviors.
Resident Intruder Test	Attack Latency (s)	The amount of time between introduction and the first clinch attack for either animal.	TBI should attack earlier and usually first	Lower attack latencies show a higher aggression if the animal attacking is the resident animal.
	Total Offense Score	The sum of lateral threat, upright standing, clinch attacking, keeping down the intruder, and chasing.	TBI animals should have higher total offense scores	A higher total offense score shows a higher level of aggression.
	Social Exploration Score	The sum of social exploration, genital sniffing, and social grooming.	TBI animals should have lower social exploration scores	A higher social exploration score shows a lower level of anxiety.
	Both above can be measured as a sum of frequencies; however, these data are usually seen as percentages of total observation time.			
Motor Coordination				
Rotarod	Latency to Fall (s)	The amount of time it takes an animal to fall off of the rotating rod.	TBI animals should perform worse during the acute phase	
Open Field Test	Total Distance Travelled (cm)	The distance the animal travels through the entire trial regardless of zone.	TBI animals should have less distance travelled. This is mainly true for the acute phase of injury.	Lower distance travelled can mean worse motor coordination. See above for the relation between total distance travelled and anxiety-like behaviors. Time after injury is an important parameter when interpreting these results.
Footprint Assay	Step Length (mm)	The distance between steps of the same paw.	Dependent on time; TBI animals should show differences during acute and subacute phases	A shorter step length in the acute and subacute phases shows poor motor coordination.
	Step Duration (ms)	The length of time one step takes.	Dependent on time; TBI animals should show differences during acute and subacute phases	A shorter step duration in the acute and subacute phases shows poor motor coordination.
	Inter-Leg Coordination	The coordination to keep both legs on each respective side within a straight line. This datum is quantitative.	Dependent on time; TBI animals should show differences during acute and subacute phases	A worse outcome of inter-leg coordination in the acute and subacute phases shows poor motor coordination.

**Table 3.** Comparison between animal models and injury severity based on injury mechanism, presence of major extracranial injury (MEI) and injury characteristics as described in the results from CENTER-TBI [13]. RTI, road traffic incident. Comparison of animal models to classifications of TBI in humans.

Injury Severity	Injury Mechanism	Presence of MEI	Imaging Characteristics	Animal Models
**Mild Upper Intermediate**	Diffuse Blunt Force Trauma Fall Sports Injury Rotational Acceleration of Brain	No	Cerebral Edema Concussion Grade 1 DAI No Presence of Lesion or Cortical Tissue Loss	CHIMERA Modified Marmarou Modified CCI Weight Drop (Marmarou) Midline FPI
**Lower Intermediate**	Fall RTI Focal Blunt Force Trauma	Possible	Diffuse Cortical Contusion Intraventricular Hemorrhage Subarachnoid Hemorrhage	Weight Drop (Shohami and Marmarou) CCI Blast Injury Lateral FPI
**Severe**	Focal Penetration Laceration GSW	Probable	Skull Fracture Focal Cortical Contusion Cortical Tissue Loss Cavity Formation Subdural Hematoma Epidural Hematoma	Weight Drop (Feeney and Shohami) CCI Lateral FPI PBBI
